# Genome-Wide Association Studies of Asthma in Population-Based Cohorts Confirm Known and Suggested Loci and Identify an Additional Association near HLA

**DOI:** 10.1371/journal.pone.0044008

**Published:** 2012-09-28

**Authors:** Adaikalavan Ramasamy, Mikko Kuokkanen, Sailaja Vedantam, Zofia K. Gajdos, Alexessander Couto Alves, Helen N. Lyon, Manuel A. R. Ferreira, David P. Strachan, Jing Hua Zhao, Michael J. Abramson, Matthew A. Brown, Lachlan Coin, Shyamali C. Dharmage, David L. Duffy, Tari Haahtela, Andrew C. Heath, Christer Janson, Mika Kähönen, Kay-Tee Khaw, Jaana Laitinen, Peter Le Souef, Terho Lehtimäki, Australian Asthma Genetics Consortium collaborators, Pamela A. F. Madden, Guy B. Marks, Nicholas G. Martin, Melanie C. Matheson, Cameron D. Palmer, Aarno Palotie, Anneli Pouta, Colin F. Robertson, Jorma Viikari, Elisabeth Widen, Matthias Wjst, Deborah L. Jarvis, Grant W. Montgomery, Philip J. Thompson, Nick Wareham, Johan Eriksson, Pekka Jousilahti, Tarja Laitinen, Juha Pekkanen, Olli T. Raitakari, George T. O'Connor, Veikko Salomaa, Marjo-Riitta Jarvelin, Joel N. Hirschhorn

**Affiliations:** 1 Respiratory Epidemiology and Public Health, Imperial College London, London, United Kingdom; 2 Department of Epidemiology and Biostatistics, Imperial College London, London, United Kingdom; 3 Department of Medical and Molecular Genetics, King’s College London, London, United Kingdom; 4 Department of Chronic Disease Prevention, National Institute for Health and Welfare, Helsinki, Finland; 5 Divisions of Genetics and Endocrinology, Children’s Hospital, Boston, Massachusetts, United States of America; 6 Broad Institute, Cambridge, Massachusetts, United States of America; 7 The Queensland Institute of Medical Research, Brisbane, Australia; 8 Division of Community Health Sciences, St George’s, University of London, London, United Kingdom; 9 MRC Epidemiology Unit, Institute of Metabolic Science, Addenbrooke’s Hospital, Cambridge, United Kingdom; 10 Department of Epidemiology and Preventive Medicine, Monash University, Melbourne, Australia,; 11 University of Queensland Diamantina Institute, Princess Alexandra Hospital, Brisbane, Australia; 12 Centre for Molecular, Environmental, Genetic and Analytic Epidemiology, University of Melbourne, Melbourne, Australia; 13 Skin and Allergy Hospital, Helsinki University Hospital, Helsinki, Finland; 14 Washington University School of Medicine, St. Louis, Missouri, United States of America; 15 Department of Medical Sciences: Respiratory Medicine and Allergology, Uppsala University, Uppsala, Sweden; 16 Department of Clinical Physiology, Tampere University Hospital, Tampere, Finland; 17 Clinical Gerontology Unit, Addenbrooke’s Hospital, Cambridge, United Kingdom; 18 Finnish Institute of Occupational Health, Oulu, Finland; 19 School of Paediatrics and Child Health, Princess Margaret Hospital for Children, Perth, Australia; 20 Department of Clinical Chemistry, University of Tampere and Tampere University Hospital, Tampere, Finland; 21 Woolcock Institute of Medical Research, University of Sydney, Sydney, Australia; 22 Wellcome Trust Sanger Institute, Wellcome Trust Genome Campus, Hinxton, United Kingdom; 23 Institute for Molecular Medicine Finland, University of Helsinki, Helsinki, Finland; 24 Medical and Population Genetics and Genetic Analysis Platform, The Broad Institute of MIT and Harvard, Cambridge, Massachusetts, United States of America; 25 Department of Medical Genetics, University of Helsinki and University Central Hospital, Helsinki, Finland; 26 Department of Children, Young People and Families, National Institute for Health and Welfare, Helsinki, Finland; 27 Institute of Clinical Medicine/Obstetrics and Gynecology, University of Oulu, Oulu, Finland; 28 Respiratory Medicine, Murdoch Children’s Research Institute, Melbourne, Australia; 29 Department of Medicine, University of Turku, Turku, Finland; 30 Helmholtz Zentrum Munchen German Research Center for Environmental Health, Munich-Neuherberg, Germany; 31 MRC Health Protection Agency (HPA) Centre for Environment and Health, Imperial College London, London, United Kingdom; 32 Lung Institute of Western Australia and Centre for Asthma, Allergy and Respiratory Research, University of Western Australia, Perth, Australia; 33 National Institute for Health and Welfare, Helsinki, Finland; 34 Unit of General Practice, Helsinki University Central Hospital, Helsinki, Finland; 35 Department of General Practice and Primary Health Care, University of Helsinki, Helsinki, Finland; 36 Folkhälsan Research Center, Helsinki, Finland; 37 Department of Pulmonary Diseases and Clinical Allergology, Turku University Hospital, Turku, Finland; 38 University of Turku, Turku, Finland; 39 Department of Environmental Health, National Institute for Health and Welfare (THL), Kuopio, Finland; 40 Institute of Public Health and Clinical Nutrition, University of Eastern Finland, Kuopio, Finland; 41 Research Centre of Applied and Preventive Medicine, University of Turku, Turku, Finland; 42 Department of Clinical Physiology, Turku University Hospital, Turku, Finland; 43 Pulmonary Center, Department of Medicine, Boston University School of Medicine, Boston, Massachusetts, United States of America; 44 The National Heart, Lung, and Blood Institute’s Framingham Heart Study, Framingham, Massachusetts, United States of America; 45 Institute of Health Sciences, University of Oulu, Oulu, Finland; 46 Biocenter Oulu, University of Oulu, Oulu, Finland; 47 Department of Genetics, Harvard Medical School, Boston, Massachusetts, United States of America; Peninsula College of Medicine and Dentistry, United Kingdom

## Abstract

**Rationale:**

Asthma has substantial morbidity and mortality and a strong genetic component, but identification of genetic risk factors is limited by availability of suitable studies.

**Objectives:**

To test if population-based cohorts with self-reported physician-diagnosed asthma and genome-wide association (GWA) data could be used to validate known associations with asthma and identify novel associations.

**Methods:**

The APCAT (Analysis in Population-based Cohorts of Asthma Traits) consortium consists of 1,716 individuals with asthma and 16,888 healthy controls from six European-descent population-based cohorts. We examined associations in APCAT of thirteen variants previously reported as genome-wide significant (*P*<5x10^−8^) and three variants reported as suggestive (*P*<5×10^−7^). We also searched for novel associations in APCAT (*Stage 1*) and followed-up the most promising variants in 4,035 asthmatics and 11,251 healthy controls (*Stage 2*). Finally, we conducted the first genome-wide screen for interactions with smoking or hay fever.

**Main Results:**

We observed association in the same direction for all thirteen previously reported variants and nominally replicated ten of them. One variant that was previously suggestive, rs11071559 in *RORA*, now reaches genome-wide significance when combined with our data (*P* = 2.4×10^−9^). We also identified two genome-wide significant associations: rs13408661 near *IL1RL1/IL18R1* (*P*_Stage1+Stage2_ = 1.1x10^−9^), which is correlated with a variant recently shown to be associated with asthma (rs3771180), and rs9268516 in the *HLA* region (*P*_Stage1+Stage2_ = 1.1x10^−8^), which appears to be independent of previously reported associations in this locus. Finally, we found no strong evidence for gene-environment interactions with smoking or hay fever status.

**Conclusions:**

Population-based cohorts with simple asthma phenotypes represent a valuable and largely untapped resource for genetic studies of asthma.

## Introduction

Asthma, characterized by episodic breathlessness, chest tightness, coughing and wheezing, is estimated to affect 300 million people worldwide [1] and is associated with morbidity and economic costs that are comparable with other common chronic diseases [2], [3]. As with many common diseases, asthma risk is determined by both genetic and environmental factors, and estimates of heritability (the proportion of variability in risk within a population due to inherited factors) range from 35 to 90% in twin studies [4], [5], [6]. However, only a handful of genetic variants have thus far been validated as associated with asthma risk at stringent levels of statistical significance.

The first genome-wide association (GWA) study for asthma [7] was conducted in 2007, with 994 child-onset asthmatics and 1,243 non-asthmatics. This study implicated a locus near *ORMDL3 –* a gene not previously suspected to have a role in asthma susceptibility. Although the initial association did not reach a widely used threshold for genomewide significance in discovery samples (*P*<5×10^−8^), the association was subsequently replicated, including numerous independent studies, particularly in European-derived and Hispanic populations [8], [9], [10], [11]. Subsequent GWA studies of asthma identified variants in *PDE4D* [12] and *DENND1B* [13] associated with asthma at genome-wide significant levels, although the proposed variant in *DENND1B* showed considerable heterogeneity of associations between different populations. In a GWA study of severe asthma, two other loci narrowly missed genome-wide significance [14]: one in the *HLA* region, and one near *RAD50*, (a locus previously shown to be associated with total IgE levels) [15]. A GWA study of eosinophil counts with follow-up testing in asthma case-control studies identified variants near *IL1RL1/IL18R1* and *IL33* as being associated with asthma [16]. More recently, a meta-analysis of 23 GWA studies by the GABRIEL consortium [17] studied 10,365 physician-diagnosed asthmatics and 16,110 participants without asthma. They observed associations with asthma, particularly childhood-onset asthma, at multiple loci (*ORMDL3/GSDMB, IL1RL1/IL18R1, HLA-DQ, IL33, SMAD3,* and *IL2RB*). Several additional loci in this study narrowly missed the genome-wide significance threshold. Most recently, GWA studies in largely non-European ancestries [18], [19] confirmed some of the loci discovered in Europeans, and identified additional associations at *PYHIN1*, *USP38-GAB1*, an intergenic region of 10p14, and a gene-rich region of 12q13. A very recent GWA study of severe asthma in Europeans found strong evidence for two previously established loci (*ORMDL3/GSDMB* and *IL1RL1/IL18R1*) in patients with severe asthma but did not identify any novel loci [20].

Many of the above findings have come from GWA studies on samples specifically established for the investigation of allergy and asthma, often with rich phenotypic information on asthma and related diseases. While such detailed phenotypic information is extremely valuable, collection of such samples is resource-intensive, potentially limiting the sample size. As for all other polygenic traits, sample sizes of current GWA studies of asthma are a limiting factor in the search for genetic risk factors [21]. Expanding GWA studies to include additional cohorts that may not have such detailed phenotypic information could increase the power to detect associations.

One route to increasing power may be found in the numerous population-based cohorts with existing genome-wide genotype data that also have basic information on doctor-diagnosed asthma, but have not been comprehensively analyzed for associations with asthma. The idea of reanalyzing existing GWA data is similar to the approach adopted by consortia studying quantitative traits that are routinely measured in many studies, such as height and weight [22], [23]. However, unlike anthropometric measures, asthma is a disease where diagnostic criteria may not always be consistent [24], [25], [26]. As such, it is uncertain whether the rather minimal phenotype of a self-report of doctor-diagnosed asthma is sufficient to be useful in genetic studies of asthma.

In this paper, we test whether a self-report of doctor-diagnosed asthma in population-based cohort could be used to replicate findings previously reported by other asthma GWA studies or identify novel genetic associations with asthma. To achieve this aim, we formed the Analysis in Population-based Cohorts for Asthma Traits (APCAT) genetics consortium, which currently includes 18,604 adults of European ancestry (1,716 cases and 16,888 controls) from six population-based cohorts with GWA data. To study further the top hits emerging from the meta-analyses of APCAT (*Stage 1*), we utilized *in-silico* replication data (*Stage 2*) from 15,286 adults of European ancestry (4,035 cases and 11,251 controls). We were able both to replicate known signals and to identify new associations, indicating that genetic studies of asthma in population-based cohorts are likely to be useful complements to more focused studies of asthma.

## Results

The APCAT genetics consortium includes 1,716 individuals with asthma diagnosed (ever) by a physician, and 16,888 non-asthmatic controls, from six population-based cohorts (**Table** **1**). Genome-wide genotyping was conducted on available platforms and, after standard quality control (see Methods), subsequently imputed to ∼2.5 million autosomal SNPs using the HapMap CEU reference panel (**Table S1**). Association studies with asthma used an additive genetic model and were adjusted for sex, ancestry-informative principal components and (in non-birth cohorts) for age; we also controlled for relatedness in family-based cohorts. We meta-analyzed the study-specific results using a fixed effect model and considered ∼2.2 million SNPs with imputation quality >0.30 and minor allele frequency >5%. We applied genomic control at the individual study level and again after meta-analysis to correct for inflation of test statistics due to any systematic bias. The individual study genomic control inflation factors were modest (λ_GC_ ≤1.02 for all studies; **Table S1**). We also explored whether stratifying the individual cohorts by smoking status or by the presence of allergic symptoms prior to meta-analysis substantially affected our results. We observed strong correspondences between the unstratified and stratified analyses (**Figure S1**), and therefore primarily report on the unstratified results as a simpler main analysis with slightly improved power.

**Table 1:** Characteristics of Stage1 and Stage2 studies.

Study	# Asthmatics cases	# Non-asthmatic Controls	Mean age at questionnaire	% male	% non-smokers	% individuals without hayfever	Genotyping platform^a^
**Stage1: APCAT studies for discovery**							
FINRISK	160	1,705	52	57.7%	47.8%	58.8%	Illumina Quad 610K (CorroGene), Affymetrix 6.0 (MIGen)
Framingham Heart Study (FHS)	797	6,463	44	43.9%	43.0%	37.7%	Affymetrix 5.0
Health 2000 (H2000)	153	1,841	49	51.1%	47.1%	67.6%	Illumina Quad 610K (GeneMets), Illumina 370K (HDL)
Helsinki Birth Cohort (HBC)	123	1,533	62	43.3%	42.3%	70.4%	Illumina 670K
Northern Finland Birth Cohort 1966 (NFBC1966)	364	3,502	31	48.2%	37.0%	64.4%	Illumina CNV370- Duo
Young Finns Study (YFS)	119	1,844	37	45.7%	49.8%	77.1%	Illumina 670K
**Total for Stage1**	**1,716**	**16,888**			**43.3%**	**55.6%**	
**Stage2: in-silico replication**							
1958 British Birth Cohort (B58C)	986	3,211	42	49.8%	28.9%	78.4%	Affymetrix 500K (WTCCC), Illumina 550K (T1DGC), Illumina Quad 610 (GABRIEL)
Australian Asthma Genetics Consortium (AAGC)^b^	2,110	3,857	34	45.2%	NA	NA	Illumina CNV 370-Duo (28%), Illumina 610K (72%)
European Community Respiratory Health Survey follow-up (ECRHS 2)	600	1,268	42	46.4%	45.7%	63.6%	Illumina Quad 610K
EPIC-Norfolk^c^ population based	216	2,005	59	46.8%	45.4%	87.9%	Affymetrix 500K
EPIC-Norfolk^c^ obese cases	123	910	60	43.0%	44.3%	87.2%	Affymetrix 500k
**Total for Stage2**	**4,035**	**11,251**					

a See **Table S1** for more information on genotyping, imputation and software used. ^b^ The characteristics of the studies in the AAGC are presented in Ferreira et al., 2011 [8]. ^c^ EPIC = European Prospective Investigation into Cancer and Nutrition.

### Analysis of genetic variants previously associated with asthma

To test whether the population-based cohorts and the phenotype of self-reported doctor-diagnosed asthma could be used to detect associations, we analyzed thirteen SNPs in nine genomic loci that had shown genome-wide significant associations in previously published GWA studies of European ancestry individuals. Encouragingly, the APCAT results (**Table** **2**) are directionally consistently for all thirteen SNPs, and nominally replicate (one-tailed *P*<0.05) ten of these thirteen SNPs. We were unable to assess the *PYHIN1* variant reported as associated with asthma in African-Americans [18] as this variant is monomorphic in European populations. For variants discovered in Japanese individuals [19], [27], it is harder to interpret replication in our samples because of differences in LD. Nevertheless, we examined the associations in APCAT for the reported lead SNPs and additional SNPs in LD in the HapMap JPT sample. For two out of the four loci, the associations showed directional consistency, with one association nominally replicated (rs1701704 in *IKZF4*, p = 0.00132, see **Table S2**).

**Table 2:** Results in APCAT for SNPs at loci with strong previously published evidence of association with asthma.

Loci with previous genome-wide significant associations to asthma (*P*<5×10^−8^)										
				Reported		APCAT		APCAT+Reported		
Gene in region^a^	SNP	Study^b^	Effect Allele^c^	OR (95% CI)	*P* value	OR (95% CI)	*P* value^d^	OR (95% CI)	*P* value^e^	
*GSDMA, GSDMB, ORMDL3*	rs3894194	1	A	1.17 (1.11,1.23)	4.6E-09	1.11 (1.04,1.18)	2.3E-03	1.15 (1.10,1.19)	1.4E-10	
	rs2305480	1	A	0.85 (0.81,0.90)	9.6E-08	0.94 (0.87,1.01)	4.1E-02	0.89 (0.84,0.93)	1.6E-07	
	rs7216389	2	T	1.18 (1.11,1.25)^f^	8.5E-08^f^	1.11 (1.04,1.19)	2.1E-03	1.15 (1.11,1.20)	2.6E-09	
*IL33*	rs3939286	3	T	1.12 (1.07, 1.17)	5.3E-06	1.18 (1.10,1.26)	4.8E-05	1.13 (1.09,1.18)	3.6E-09	
	rs1342326	1	C	1.20 (1.13,1.28)	9.2E-10	1.18 (1.09,1.28)	2.5E-04	1.2 (1.15,1.25)	2.0E-12	
*HLA-DQ*	rs9273349	1	C	1.18 (1.13,1.24)	7.0E-14	1.22 (1.07,1.38)^g^	5.6E-03^g^	1.19 (1.14,1.23)	2.8E-15	
*IL18R1, IL1RL1*	rs3771166	1	A	0.87 (0.83,0.91)	3.4E-09	0.89 (0.82,0.97)	2.4E-03	0.88 (0.84,0.92)	7.0E-11	
	rs1420101	3	T	1.16 (1.11, 1.21)	5.5E-12	1.16 (1.08,1.23)	9.9E-05	1.16 (1.12,1.20)	4.9E-15	
*SMAD3*	rs744910	1	A	0.89 (0.86,0.92)	3.9E-09	0.92 (0.85,1.00)	1.7E-02	0.90 (0.86,0.93)	5.7E-10	
*IL2RB*	rs2284033	1	A	0.89 (0.86,0.93)	1.2E-08	0.98 (0.91,1.06)	3.1E-01	0.91 (0.88,0.95)	1.4E-07	
*IL13*	rs1295686^I^	1	C	0.87 (0.83,0.92)	1.4E-07	0.90 (0.82,0.98)	5.4E-03	0.88 (0.84,0.92)	5.9E-09	
*DENND1B*	rs2786098	4	T	0.70 (0.63,0.78)	3.9E-11	0.93 (0.89,1.02)	5.8E-02	0.83 (0.76,0.90)	4.7E-08	
*PDE4D*	rs1588265	5	G	0.85 (0.87,0.93)	2.5E-08	0.96 (0.85,1.06)	2.0E-01^h^	0.87 (0.82,0.92)	1.1E-07	
**Loci with previous suggestive associations to asthma (** ***P*** **<5×10^−7^)**										
*RORA*	rs11071559	1	T	0.85 (0.80,0.90)	1.1E-07	0.86 (0.75,0.97)	3.1E-03	0.85 (0.80,0.91)		2.4E-09
*RAD50*	rs2244012	6	G	1.64 (1.36,1.97)	3.0E-07	1.05 (0.96,1.14)	1.3E-01	1.14 (1.06,1.22)		1.5E-03
*SLC22A5*	rs2073643	1	C	0.90 (0.87,0.94)	2.2E-07	0.96 (0.88,1.04)	1.6E-01	0.91 (0.88,0.95)		3.9E-07

a Gene shown is nearest gene to associated SNP. SNPs from the same locus are grouped together. ^b^References: 1 = Moffatt et al. (2010) [17]; 2 = Moffatt et al. (2007) [7]; 3 = Gudbjartsson et al. (2009) [16]; 4 = Sleiman et al (2010) [13]; 5 = Himes et al (2009) [12]; 6 = Li et al. (2010) [22].^ c^Alleles are indexed to the forward strand of NCBI build36. ^d^APCAT *P* values are one-tailed with respect to the direction of the original association.^ e^ *P* values are from fixed-effect inverse-variance model of meta analysis. ^f^Results shown are from Moffatt et al (2010), which is the larger and more recent study. ^g^ SNP rs9273349 is present in NFBC1966 data set only. ^h^Results exclude the Framingham Heart Study, which contributed to the original report in Himes et al (2009) ^I^Shown here are the random effects *P* value in Gabriel data, the *P* value for fixed effects model had a genome wide significance *P* value of 1.4E-08 with no evidence of heterogeneity.

We also examined three SNPs where previous evidence was strongly suggestive (*P*<5×10^−7^ for association with asthma) but not genome-wide significant (**Table** **2**), and one of them, rs11071559 in *RORA,* is strongly supported in our data (Reported *P* = 1.1x10^−7^; *P_APCAT_*  = 0.0031). The combined evidence of association at *RORA* (*P* = 2.4×10^−9^) now surpasses the genome-wide significance threshold, and therefore rs11071559 represents a new genome-wide significant association with asthma. We note that the *RORA* locus is 6.4 Mb away from a known asthma variant (rs744910 near *SMAD3*), but these two loci are independent, because there is low linkage disequilibrium between these two variants (pairwise linkage disequilibrium, *r* ^2^ = 0.01 in HapMap CEU panel) and because the association estimates for these two variants are virtually unchanged when conditioned on each other (**Table S3**).

### Search for novel asthma risk loci in APCAT

Having established the validity and utility of our population-based studies, we next examined the results of a genome-wide meta-analysis of the studies within APCAT (**Figure S2**), with the goal of identifying novel associations with asthma. An inspection of the quantile-quantile plots (**Figure S2**) and the low genomic inflation factor (λ = 1.01) of the meta-analyses indicate that there is little evidence of confounding by population stratification or other technical artifacts.

To test whether some of the top results from the APCAT analysis could represent valid associations with asthma, we considered the most strongly associated 14 SNPs from independent loci within the APCAT results (*Stage1* in **Table** **3**). We obtained *in silico* replication data for 14 top SNPs from several additional studies of asthma, including two population based cohorts. These replication studies consisted of the 1958 British Birth Cohort (B58C), Australian Asthma Genetics Consortium (AAGC), the second survey of the European Community Respiratory Health Survey (ECRHS2) and the European Prospective Investigation of Cancer in Norfolk (EPIC-Norfolk) (**Table S1**). The results from the replication studies (*Stage2*) and a meta-analysis of these results with the APCAT data (*Stage 1* + *Stage 2*) are summarized in **Table** **3**.

**Table 3:** Replication results for top signals from APCAT (Stage1 *N* = 18,604) in additional studies (Stage 2 *N* = 15,576) and in GABRIEL.

Nearest Gene(s)	SNP	Chr (position^a^)	Effect/alternate allele^a^	Effect allele frequency	Stage1 (APCAT) OR (95% CI) and *p* value	Stage2 (replication) OR (95% CI) and *p* value	Stage1 + Stage2 (pooled) OR (95% CI) and *p* value	GABRIEL^b^ fixed-effects OR (95% CI) and *p* value	GABRIEL SNP (*r* ^2^ in HAPMAP CEU with APCAT SNP)^c^
*IL1RL1/IL18R1*	rs13408661	2 (102321514)	G/A	0.84	1.29 (1.16,1.44); *P = *3.9E-06	1.19 (1.10,1.29); *P = *3.2E-05	1.23 (1.15,1.31); *P = *1.1E-09	1.15 (1.09,1.22); *P = *7.0E-06	rs3213733 (*r* ^2^ = 0.83)
*BTNL2/HLA-DRA*	rs9268516	6 (32487467)	T/C	0.24	1.26 (1.17, 1.37); *P = *1.2E-07	1.11 (1.04,1.17); *P = *1.0E-03	1.15 (1.10,1.21); *P = *1.1E-08	1.05 (0.99,1.12); *P = *6.9E-02	rs8180664 (*r* ^2^ = 0.99)
*IFNE*	rs7861480	9 (21460997)	T/C	0.11	1.28 (1.17,1.39); *P = *9.8E-06	1.09(1.00,1.18); *P = *5.7E-02	1.17 (1.10,1.24); *P = *1.7E-05	1.04 (0.96,1.12); *P = *1.9E-01	rs10811568 (*r* ^2^ = 0.87)
*CSMD1*	rs2977724	8 (4911545)	T/C	0.88	1.34 (1.21,1.46); *P = *5.6E-06	1.07 (0.99,1.15); *P = *1.0E-01	1.14 (1.08,1.21); *P = *1.2E-04	0.98 (0.90,1.05); *P = *7.4E-01	Same SNP
*SLC6A15/TMTC2*	rs4882411	12 (83141816)	A/C	0.76	1.22 (1.14,1.31); *P = *7.3E-06	1.05 (0.99,1.15); *P = *1.0E-01	1.11 (1.06,1.16); *P = *8.3E-05	1.03 (0.98,1.08); *P = *1.5E-01	rs1564606 (*r* ^2^ = 0.91)
*ATP2B2*	rs26310	3 (10347840)	T/C	0.67	1.22 (1.13,1.30); *P = *7.1E-06	1.05 (0.99,1.11); *P = *9.2E-02	1.10 (1.05,1.15); *P = *7.7E-05	1.01 (0.96,1.06); *P = *3.2E-01	Same SNP
*DACH1/C13orf37*	rs1460456	13 (71402712)	G/A	0.21	1.22 (1.13,1.31); *P = *1.5E-05	1.02 (0.93,1.04); *P = *7.0E-01	1.10 (1.04,1.16); *P = *1.7E-03	1.03 (0.96,1.09); *P = *2.2E-01	Same SNP
*ZNF479/MIR3147*	rs10227804	7 (57341784)	G/A	0.48	1.19 (1.12,1.26); *P = *2.5E-06	0.98 (0.93,1.04); *P = *5.8E-01	1.06 (1.02,1.11); *P = *1.1E-02	0.99 (0.93,1.05); *P = *5.9E-01	rs1403937 (*r* ^2^ = 0.95)
*TRERF1*	rs4714586	6 (42352608)	A/G	0.41	1.18 (1.11,1.25); *P = *9.3E-06	0.98 (0.92,1.03); *P = *4.2E-01	1.05 (1.00,1.09); *P = *3.9E-02	0.99 (0.94,1.04); *P = *6.7E-01	Same SNP
*SPRY1/ANKRD50*	rs2553377	4 (170622584)	A/C	0.20	1.23 (1.13,1.32); *P = *2.4E-05	0.97 (0.89,1.04); *P = *3.8E-01	1.06 (1.00,1.12); *P = *5.2E-02	1.01 (0.95,1.08); *P = *3.4E-01	Same SNP
*MECOM*	rs1918969	3 (170622584)	T/C	0.55	1.18 (1.11,1.26); *P = *9.0E-06	0.99 (0.93,1.04); *P = *6.4E-01	1.05 (1.01,1.10); *P = *2.4E-02	1.00 (0.95,1.05); *P = *5.4E-01	rs4245909 (*r* ^2^ = 0.78)
*NEDD4L*	rs292448	18 (54047200)	A/C	0.69	1.21 (1.12,1.29); *P = *1.2E-05	0.98 (0.92,1.04); *P = *5.4E-01	1.05 (1.00,1.10); *P = *3.7E-02	1.02 (0.97,1.07); *P = *2.7E-01	rs292451 (*r* ^2^ = 0.96)
*SLC7A11/PCDH18*	rs6825001	4 (139122321)	G/A	0.10	1.27 (1.15, 1.39); *P = *8.1E-05	1.06 (0.97,1.15); *P = *2.3E-01	1.05 (0.98,1.12); *P = *1.6E-02	0.90 (0.81,0.98); *P = *9.9E-01	Same SNP
*GPD1L/CMTM8*	rs7620066	3 (32194428)	T/A	0.35	1.14 (1.06, 1.22); *P = *8.7E-04	0.95 (0.89,1.01); *P = *8.3E-02	1.02 (0.97,1.06); *P = *5.3E-01	1.04 (0.99,1.09); *P = *7.9E-02	rs7644491 (*r* ^2^ = 0.84)

a Positions/alleles are relative to the forward strand of NCBI build36. ^b,c^Results from GABRIEL are from a re-analysis using fixed-effects meta-analysis, excluding the B58C and ECRHS2 cohorts which are included in Stage2 or with occupational asthma (see Methods), and are for the APCAT SNP or the best available proxy. All p values are two-tailed.

**Figure 1:**
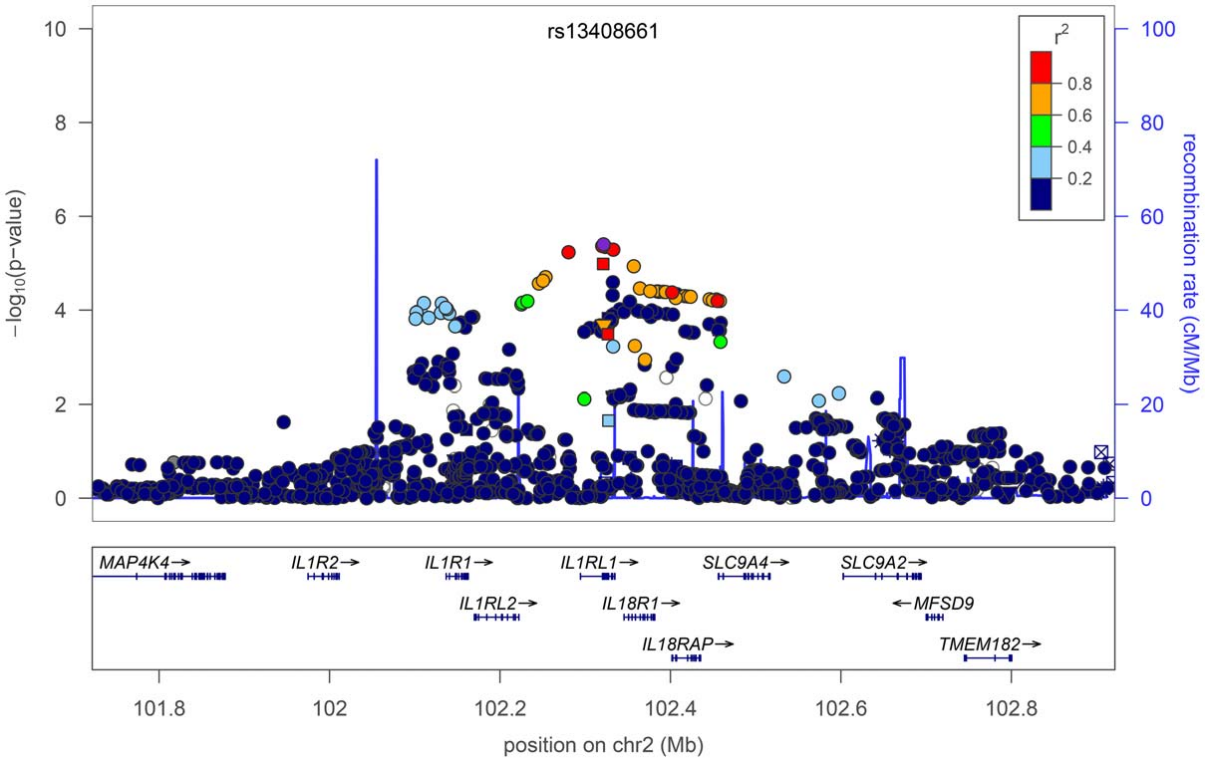
Regional association and forest plots for rs13408661 in the *IL1RL1/IL18R1* locus. For the regional plot, the lead SNP is indicated by a purple diamond, and the degree of linkage disequilibrium (*r* ^2^) of other SNPs in the region to the lead SNP is indicated by the color scale. Genes are shown below, and estimated recombination rate is indicated by the blue lines. Note that the regional plot is based on Stage1 (pooled) estimates only. For the forest plot, the estimated odds ratio and 95% confidence interval for each individual study is shown by the boxes (scaled to sample size) and lines; pooled estimates and 95% confidence intervals are indicated by diamonds.

The most strongly replicated SNP is rs13408661 near the *IL1RL1* and *IL18R1* genes, with the combined *P* value reaching genome-wide significance (*P*_Stage1_  = 3.9×10^−6^; *P*_Stage2_  = 3.2×10^−5^; *P*_Stage1+Stage2_  = 1.1×10^−9^; **Figure** **1**). This SNP lies approximately 31.1 kb from rs3771166 (pairwise *r* ^2^ = 0.157) identified by GABRIEL [17] and 2.6 kb from rs1420101 (*r* ^2^ = 0.053) identified by an earlier study [16] as genetic risk factors for asthma. Conditioning rs13408661 on either rs3771166 or rs1420101 did not substantially reduce the signal of association in APCAT (**Table S3**), implying that rs13408661 represents a different signal of association at this locus. Interestingly, a previous study focusing on previously reported risk loci for asthma in a subsample of the Australian samples in this report [8] had also suggested that an association with rs10197862, which is tightly correlated with rs13408661 (*r* ^2^ = 0.932), was independent of other associated variants in the region. Quite recently, a study of ethnically diverse sample of Americans [18] identified a genome-wide significant association at rs3771180/rs10173081, which are also tightly correlated with rs13408661 (*r* ^2^ = 0.907 and 1). Therefore, the combined data, including our conditional analyses, indicate that rs13408661 truly represents an additional genome-wide significant signal of association with asthma at this locus.

**Figure 2:**
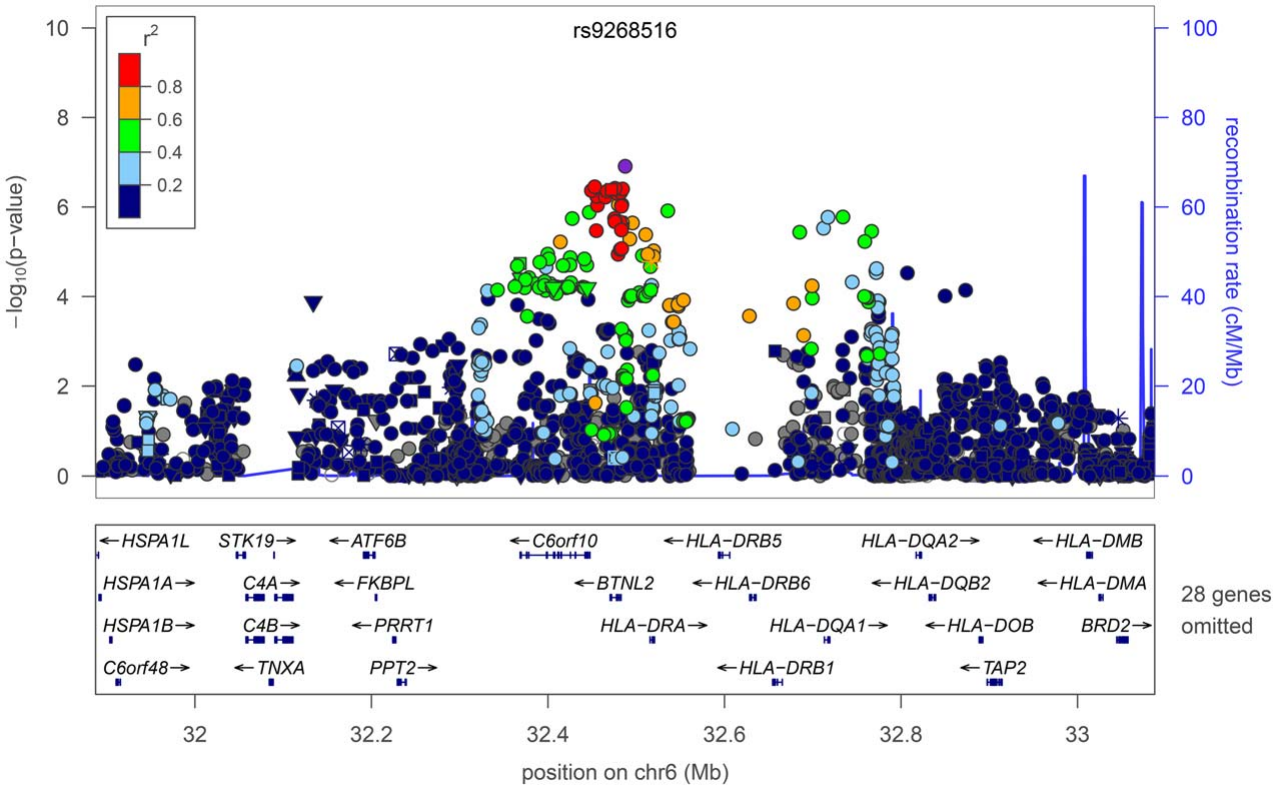
Regional association and forest plots for rs9268516 in the HLA region, with symbols as in **Figure 1**.

Our second strongest signal is at rs9268516 (*P*_Stage1_  = 1.2×10^−7^; *P*_Stage2_  = 1.0×10^−3^; *P*_Stage1+Stage2_  = 1.1×10^−8^; **Figure** **2**) in the HLA region on Chromosome 6, approximately 246 kb away from the rs9273349 variant (*r* ^2^ = 0.324) identified by GABRIEL [17]. Conditional analysis (**Table S3**) indicates that the association at rs9273349 cannot completely explain the association at rs9268516, suggesting either the presence of multiple signals at this locus or the presence of a third variant (partially correlated with both rs9273349 and rs9268516) that explains the association at both of these variants.

None of the other variants had compelling evidence of replication in our *Stage2* data (**Table** **3**), although rs7861480 near *IFNE* showed a trend in the same direction (*P* = 0.064). We also examined the evidence for association of these 14 SNPs or their proxies in the GABRIEL data (estimates downloaded from http://www.cng.fr/gabriel/results.html), excluding B58C and ECRHS (which participated in *Stage2* of the APCAT study) and the occupational asthma cohorts. Consistent with our *Stage2* results, we saw directionally consistent evidence of association at rs13408661 (*P* = 7.0×10^–6^) in the *IL1RL1/IL18R1*locus and rs9268516 in the *HLA* region (*P* = 0.069); rs7861480 near *IFNE* showed directional consistency but was not significantly associated with asthma in GABRIEL (*P* = 0.19).

We also estimated the variance explained in the APCAT by the most strongly associated SNP at each of the previously reported loci. Under a liability threshold [28], and assuming a prevalence of 9% (the prevalence in APCAT), these SNPs together explain ∼1.6% of the population variance in asthma risk (**Table** **S4**). Of course, additional variance is explained by multiple signals at each locus (such as at IL1RL1/IL18R1and HLA regions), variants at additional loci (such as at RORA), and variants yet to be discovered by additional genetic studies.

### Search for interactions with smoking and allergic status in APCAT

To our knowledge, there has been no genome-wide search for gene-environment interactions with smoking status or allergic status, two important modifiers for risk of developing asthma. We scanned ∼2.2 million SNPs for gene-environment effects for smoking exposure in APCAT by comparing, for each SNP, the estimated association statistics with asthma in current smokers to the estimates in never smokers. The strongest evidence for interaction with smoking status did not reach genome-wide significance [estimated pooled odds ratio for interaction between rs1007026 (nearest genes are MOCS1 and DAAM2) and smoking status  = 1.89; 95% confidence interval 1.43 to 2.49, *P* = 8.6×10^−6^; **Figure S3**]. Similarly, we scanned for interactions with allergic status in APCAT by comparing, for each SNP, the estimated association statistics for asthma in individuals with hay fever (the most commonly available measure of allergic risk factors in the APCAT studies) to the estimates in individuals without hay fever. The locus with the strongest evidence of interaction with allergic status did not reach genome-wide significance either [estimated pooled odds ratio for interaction between rs17136561 (located in SLC22A23 which overlaps with PSMG4 and TUBB2B) and hay fever status  = 1.64; 95% confidence interval 1.33 to 2.02, *P* = 2.3×10^−6^; **Figure S3**]. We did not pursue either of these loci further.

We also investigated whether the associations of the previously known or suggestive loci, and the signals emerging from this paper (rs13408661 near the *IL1RL1/IL18R1* genes and rs9268516 in the HLA region), differed in association by smoking or allergic status. The direction of association for asthma in the never smokers and also in non-allergic individuals (*i.e.* the healthy subgroups) were generally consistent with the unstratified analysis, but with weaker signals as expected with reduced sample sizes (**Table S5**). A formal test for heterogeneity between smoking strata indicated no significant differences. Similarly, there was no significant heterogeneity for the allergic strata except possibly for rs2284033 in *IL2RB* (*P*_heterogeneity_  =  0.038), where opposite directions of association with asthma were observed in the two allergic strata, and for rs11071559 in *RORA* (*P*_heterogeneity_  = 0.059) where the signal for asthma association appears to be seen predominantly in the allergic individuals.

## Materials and Methods

### Participants and Studies

Cases and controls for the discovery study were drawn from six population-based studies of individuals of European ancestry: FINRISK [29], Framingham Heart Study [30], Health 2000 [31], Helsinki Birth Cohort [32], Northern Finland Birth Cohort of 1966 [33] and Young Finns Study [34]. All cohorts were genotyped using commercially available genotyping arrays and SNPs which passed QC filters were used to impute up to 2.5 million SNPs using HapMap CEU as the reference. Participants for the *in silico* replication were drawn from the 1958 British Birth Cohort (B58C) [7], the Australian Asthma Genetics Consortium (AAGC) [35], European Community Respiratory Health Survey followup (ECRHS) [36] and EPIC-Norfolk [37]. Study characteristics are given in **Table** **1** and **Supplementary Methods**; genotyping and imputation details are given in **Table S1**. The most strongly associated SNPs from APCAT were checked for validity after re-analysis of data from the GABRIEL (A Multidisciplinary Study to Identify the Genetic and Environmental Causes of Asthma in the European Community) study [17] (excluding B58C, ECRHS2 and cohorts with occupational asthma) made available at www.cng.fr/gabriel (Table 3).

### Phenotype definition and stratification

Cases were defined as individuals who had given an affirmative questionnaire response to the question “Have you ever been diagnosed with asthma?” (exact wording varied among questionnaires – see **Supplementary Methods**). The remaining subjects served as healthy controls if they did not affirmatively respond to any of the following: self-reported asthma without a physician diagnosis, chronic obstructive pulmonary disease, emphysema, chronic bronchitis, chronic cough associated with wheeze, other lung disease, or FEV_1_ <70% of predicted. Individuals with reports of chronic obstructive pulmonary disease, emphysema, chronic bronchitis, or other lung diseases were also excluded from the cases.

We also conducted two stratified analyses of asthma: smoking-stratified and allergy-stratified. Allergic status was defined using an affirmative response to the question “Have you ever had hay fever or other allergic nasal symptoms?” (exact wording varied among questionnaires), as this was the most uniformly available information on allergy in APCAT. Participants were divided into three smoking categories: never smokers, ex-smokers if smoked regularly more than a year ago or current smokers if currently smoking or smoked regularly in past year.

### Statistical analyses

The association statistic for each SNP oriented towards the forward strand and was calculated assuming an additive genetic model adjusting for sex, ancestry-informative principal components, and (in non-birth cohorts) for age. In the family-based Framingham Heart Study, the association analysis was done controlling for family structure using the GWAF package in R [38]. The data for FINRISK, Health 2000, the Helsinki Birth Cohort, and the Young Finns Study were analyzed together with an adjustment term for cohort. The data from this combined dataset along with data from the Framingham Heart Study and the Northern Finland Birth Cohort of 1966 were meta-analyzed and verified using the fixed effect inverse-variance method implemented in METAL [39] and R. Genomic control was applied at the individual study level as well as in meta-analysis stage. Approximately 2.2 million SNPs with imputation quality (info) >0.30 and minor allele frequency (MAF) >5% were analyzed.

### SNP selection for *in silico* replication

We selected a locus (defined as a region 500 kb wide) for further follow-up if it either contained a single SNP with *P*<10^−6^ or multiple SNPs with *P*<10^−5^. The “sentinel SNP” was defined as the SNP with the most significant *P* value and was included in the replication list.

## Discussion

We completed a genome-wide association study of a simple asthma phenotype – self-report of ever having been diagnosed by a physician with asthma – from 18,604 participants from six population-based studies comprising the APCAT consortium. We performed follow-up analyses of the top signals from APCAT in 15,286 additional individuals. These results provided strong evidence for an additional associated variant in the *HLA* region, a known asthma locus, and confirmed recent reports of multiple associations at the *IL1RL1/IL18R* locus. We also examined the evidence for association in APCAT of SNPs with previous genome-wide or suggestive evidence of asthma, and show that the results from our population-based studies validate and in one case newly establish genome-wide significant associations with asthma. Finally, we found no evidence of genes modifying the relation between smoking and asthma or the relationship between hay fever and asthma.

The present study has several strengths. First, it demonstrates the usefulness of a large untapped resource to complement genetic studies of asthma: population-based studies with genome-wide genotype data and a simple asthma phenotype: self-reported information on doctor-diagnosed asthma. We also present the first comprehensive search for genetic interactions with smoking status and with hay fever, two important modifiers of asthma development. Finally, we provide evidence for new genome-wide significant associations with asthma: one novel signal where there was prior suggestive evidence of association (*RORA*), one independent novel signal at a previously associated locus (the *HLA* region), and one previously associated locus where we demonstrate multiple independent signals (*IL1RL1/IL18R1*).

It is important to recognize some limitations of this present study. First, the constituent studies in APCAT studies have limited information on asthma, such as age of asthma onset or severity of symptoms, which prevents a more detailed investigation of associated loci. Second, although controls were carefully selected to exclude individuals with other respiratory diseases or abnormalities on spirometry (see methods) that may share pleiotropic risk alleles with asthma, the choice of controls in population-based cohorts is potentially subject to misclassification bias due to the inclusion of undiagnosed cases among the controls. We note that this problem is common to many study designs for diseases with variable age at onset such as asthma, but does not prevent the identification of new disease markers. Third, the power to detect novel associations in population based studies is restricted by the low prevalence of disease (our prevalence was ∼9%) compared to a case-control study of an equivalent total sample size and an equal number of cases to controls. However, we note that the large number of additional available population-based studies similar to the ones in APCAT still represent a large untapped pool of genotyped cases. Fourth, we only examined SNPs with frequencies above 5%, so have not tested rarer variants for association to asthma. Finally, the use of self-reported diagnosis of asthma in the APCAT cohorts, even though it was doctor-diagnosed, may also have led to misclassification within cases.

Despite these limitations, we were able to independently validate 10 of 13 SNPs previously reported as being associated for asthma through GWA studies, which indicates that population-based studies with simple asthma phenotypes can indeed complement ongoing genetic studies in asthma. For most (but not all) of these variants, the estimated effect sizes in APCAT are smaller than reported, which could be due to the “winner’s curse” phenomenon [40], slightly greater misclassification in our cohorts, or other differences between this study and previous reports. However, the fact that most of the APCAT odds ratios fall within the 95% confidence intervals of the original reports and the p-values typically becomes more significant when combined with the APCAT data suggests that the power gained by adding population-based studies still outweigh the effects of any misclassification there may be. When the reported estimates are combined with our data, the variant rs11071559 in *RORA* reaches genomewide significance. In the subsequent analysis, the association in *RORA* was perhaps more strongly associated with asthma in individuals with hay fever. This gene belongs to a subfamily of nuclear orphan receptors suggested to negatively regulate inflammatory response [41]. Interestingly, *RORA* deficient mice have diminished capacity to mediate allergic inflammatory response [41], [42], [43]. A recent paper found that *RORA* is critical for the development of nuocytes, which are part of the innate immune response and contribute to asthma response, in mice [44] Another recent study that looked at asthma candidate genes found that *RORA* is differentially expressed during lung development in both mouse and human [45].

By analyzing the results of meta-analysis of APCAT studies, we identified and successfully replicated associations of rs13408661 in the *IL1RL1/IL18R1* region and of rs9268516 in the HLA region; the association with rs13408661 is in agreement with recent findings [8], [18]. These two loci had originally been reported to contain other strongly associated variants [16], [17], which we now show are distinct from the ones we identify. Observing multiple signals at a locus could either be due to multiple true causal variants, as seen in genetic studies for height and other polygenic traits [22], [46], or due to a single causal variant that is not well tagged by either signal. While fine mapping of data in these regions would be required to resolve this problem conclusively, we note that the variants we identified were only modestly correlated with published variants and remain significant even after conditioning on known risk variants. Thus, our data provide two genome-wide significant signals of association at known loci that are distinct from the first variants originally reported to be associated with asthma at genome-wide significance, and in one case (*HLA*) is not accounted for by the known associations.

Finally, we present the first comprehensive search for genetic interactions with smoking status and with hay fever, two potentially important modifiers of asthma development. In our studies, we found no convincing evidence for SNPs that interacted with either smoking or hay fever. A larger meta-analysis would be required to confirm the absence or existence of such gene-environment effects for asthma.

In conclusion, these results strongly suggest that GWA studies of population-based cohorts with simple asthma phenotypes are an effective approach to find novel asthma-associated variants, replicate signals identified in other studies, and provide estimates that are representative of the demography and disease spectrum. Such cohorts are an untapped resource that can be utilized to complement genetic studies of asthma. We anticipate that many more population-based studies could be leveraged to assist with discovery of asthma susceptibility loci, which could potentially lead to more effective or targeted therapies and preventions.

## Supporting Information

Figure S1 **Correlation between unstratified analysis and smoking or allergic-status stratified analyses.**
(TIF)

Figure S2 **Quantile-quanitle plot and Manhattan plot for meta-analyses of asthma (basic unstratified analysis) in APCAT.** The shaded area in the quantile-quantile plot shows the 95% confidence intervals.
(TIF)

Figure S3 **Manhattan plot for interaction effect for smoking exposure and for allergic status and a graphical depiction of the odds ratios for the best signals.**
(TIF)

Supplementary Methods S1 **Supplementary Methods and Materials.**
(DOCX)

Table S1 **Genotyping platform, calling algorithm, imputation details and software used in Stage 1 and Stage 2 studies.**
(DOCX)

Table S2 **Loci with previous genome-wide significant associations to asthma (P<5x10^−8^) in Japanese populations.**
(DOCX)

Table S3 **Conditional analysis on novel signals in APCAT**.
(DOCX)

Table S4 **Variance explained by the GWAs hits under a liability threshold model.**
(DOCX)

Table S5 **Stratified association analysis of previously published SNPs and two novel loci emerging from this paper**.
(DOCX)
