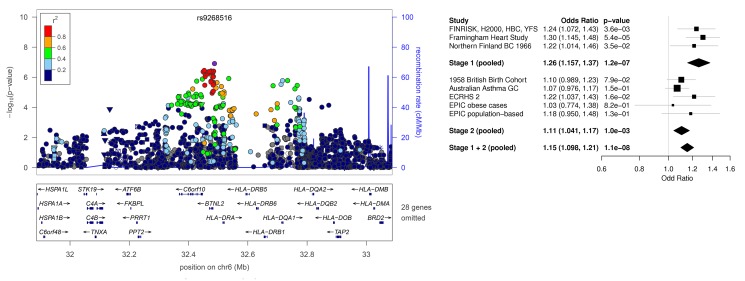# Correction: Genome-Wide Association Studies of Asthma in Population-Based Cohorts Confirm Known and Suggested Loci and Identify an Additional Association near HLA

**DOI:** 10.1371/annotation/9630862b-4676-4b82-9869-8d8fbb2a2e65

**Published:** 2013-03-04

**Authors:** Adaikalavan Ramasamy, Mikko Kuokkanen, Sailaja Vedantam, Zofia K. Gajdos, Alexessander Couto Alves, Helen N. Lyon, Manuel A. R. Ferreira, David P. Strachan, Jing Hua Zhao, Michael J. Abramson, Matthew A. Brown, Lachlan Coin, Shyamali C. Dharmage, David L. Duffy, Tari Haahtela, Andrew C. Heath, Christer Janson, Mika Kähönen, Kay-Tee Khaw, Jaana Laitinen, Peter Le Souef, Terho Lehtimäki, Australian Asthma Genetics Consortium collaborators, Pamela A. F. Madden, Guy B. Marks, Nicholas G. Martin, Melanie C. Matheson, Cameron D. Palmer, Aarno Palotie, Anneli Pouta, Colin F. Robertson, Jorma Viikari, Elisabeth Widen, Matthias Wjst, Deborah L. Jarvis, Grant W. Montgomery, Philip J. Thompson, Nick Wareham, Johan Eriksson, Pekka Jousilahti, Tarja Laitinen, Juha Pekkanen, Olli T. Raitakari, George T. O'Connor, Veikko Salomaa, Marjo-Riitta Jarvelin, Joel N. Hirschhorn

The forest plots are missing from Figures 1 and 2. The complete images for Figures 1 and 2 can be seen here:

Figure 1: 

**Figure:**
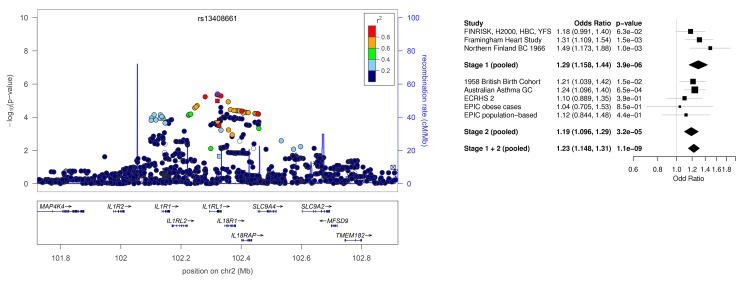


Figure 2: 

**Figure:**