# Supplementary figures and images for: Genome-Wide Association Studies of Asthma in Population-Based Cohorts Confirm Known and Suggested Loci and Identify an Additional Association near HLA
Source: PLoS One. 2012 Sep 28;7(9):e44008. doi: 10.1371/journal.pone.0044008 (PMC3461045; doi:10.1371/journal.pone.0044008)

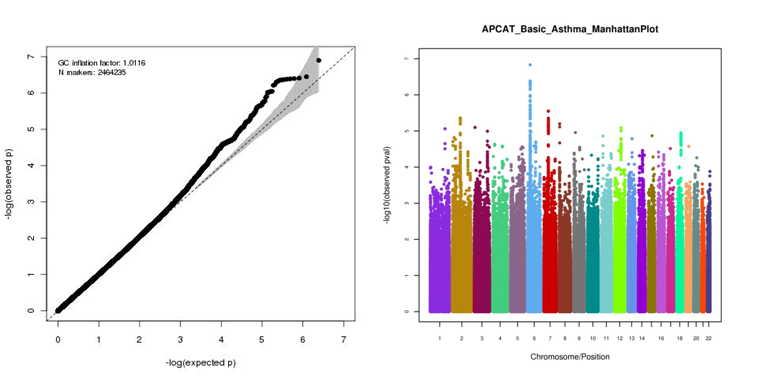

Supplement: Figure S2 — Quantile-quanitle plot and Manhattan plot for meta-analyses of asthma (basic unstratified analysis) in APCAT. The shaded area in the quantile-quantile plot shows the 95% confidence intervals. (TIF) [file pone.0044008.s002.tif]

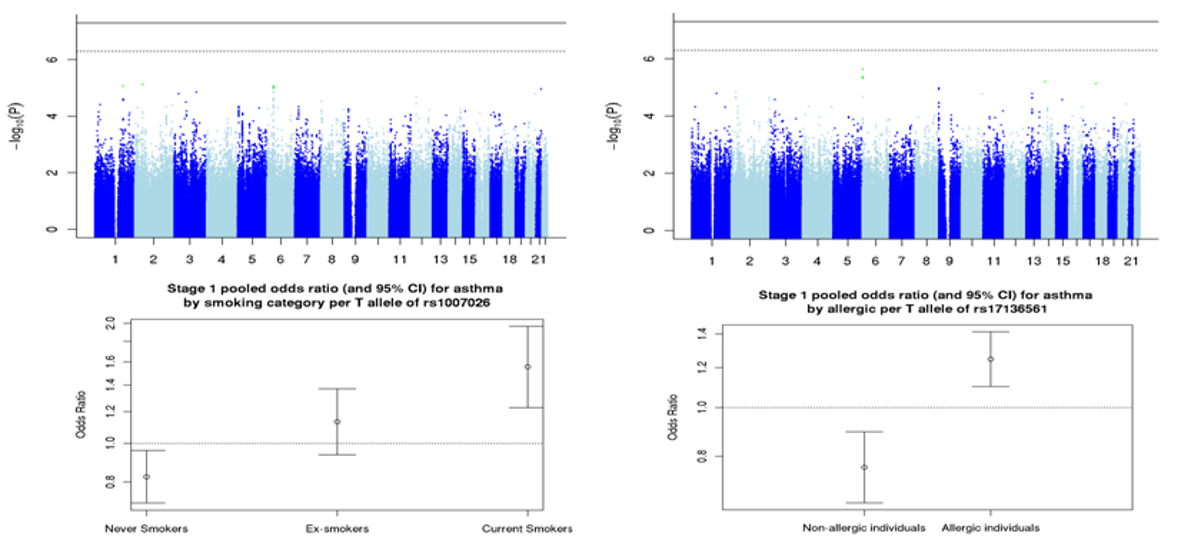

Supplement: Figure S3 — Manhattan plot for interaction effect for smoking exposure and for allergic status and a graphical depiction of the odds ratios for the best signals. (TIF) [file pone.0044008.s003.tif]
