# Supplementary material for: Genome-Wide Association Studies of Asthma in Population-Based Cohorts Confirm Known and Suggested Loci and Identify an Additional Association near HLA
Source: PLoS One. 2012 Sep 28;7(9):e44008. doi: 10.1371/journal.pone.0044008 (PMC3461045; doi:10.1371/journal.pone.0044008)
Supplement: Supplementary Methods S1 — Supplementary Methods and Materials. (DOCX) [file pone.0044008.s004.docx]

**SUPPLEMENTARY METHODS**

**Genome-wide association studies of asthma in population-based cohorts confirm known and suggested loci and identify an additional association in the HLA region**

**Stage 1 – Brief study description, funding acknowledgement and phenotype definition**

**FINRISK STUDY**

*Description:* A population survey of risk factors for chronic diseases in Finland. The survey has been executed every five years from 1972 using independent, random and representative population samples from five geographical areas of the country. Participants have filled in a health-related questionnaire and undergone a physical examination including measurement of anthropometric traits and blood draw (E[1](#_ENREF_1)).

*Funding:* Mainly funded by the Finnish National Institute for Health and Welfare with additional financial support from the Academy of Finland (grant number 129494, 139635), the Finnish Foundation for Cardiovascular Research, and the Sigrid Juselius Foundation. Participants were genotyped as part of the COROGENE (Genetic Predisposition of Coronary Heart Disease in Patients Verified with Coronary Angiogram) (E[2](#_ENREF_2)) and MIGen (Myocardial Infarction Genetics Consortium) (E[3](#_ENREF_3)) efforts.

Asthma status: For the FINRISK 1992 cohort, individuals who have answered “YES” to the question “***Have you had any of the following diseases diagnosed or treated by a doctor during the past year (last 12 months)?:*** Asthma of the lungs”. were considered to have asthma. For the FINRISK 1997, 2002 and 2007 cohorts, information about asthma was based on the following question: “***Have you ever been diagnosed with asthma?”* Participants responding “YES” were considered to have asthma.** In all FINRISK surveys the rest of the participants served as controls if their age was less than or equal to 70 years and they did not report pulmonary emphysema, or chronic bronchitis during the last 12 months.

*Smoking status:* Participants were considered as never smokers if they a**nswered “NO” to the question *“Have you ever smoked?”*** and as ex smokers if they had quit smoking at least a month ago. Current smokers were participants who smoked regularly at the time of the survey or had been smoking regularly less than 1 month ago.

*Allergy status:* **Information about allergy was based on the question *“Have you ever had hay fever of other allergic nasal symptoms?”* Participants responding “YES, during the last 12 months”, and those responding “YES, over a year ago” were considered to have allergy.**

***Framingham Heart Study (FHS)***

*Description:* Began in 1948 by recruiting an Original Cohort of 5,209 men and women between the ages of 30 and 62 from the town of Framingham, Massachusetts, who had not yet developed overt symptoms of cardiovascular disease or suffered a heart attack or stroke (E[4](#_ENREF_4)). Since that time the study has added an Offspring Cohort of 5,124 individuals in 1971 and a Third Generation Cohort of 4,095 individuals in 2002 (E[5](#_ENREF_5), [6](#_ENREF_6)). The Offspring Cohort is made up of the offspring of the members of the Original Cohort and their spouses, and the Third Generation Cohort is made up of the children of the Offspring cohort. Individuals in the FHS had exams every two years consisting of a detailed medical history, physical examination, and laboratory tests. We used data from the exam questions described below for the purposes of determining asthma cases and controls and to determine smoking and allergy statuses.

*Funding:* Supported by the National Heart, Lung and Blood Institute's Framingham Heart Study (Contract No. N01-HC-25195) and its contract with Affymetrix, Inc for genotyping services (Contract No. N02-HL-6-4278). A portion of this research utilized the Linux Cluster for Genetic Analysis (LinGA-II) funded by the Robert Dawson Evans Endowment of the Department of Medicine at Boston University School of Medicine and Boston Medical Center.

*Asthma status:* For the Original Cohort, data from Exams 1 and 5 were used. Individuals who answered “yes” to any asthma question were coded as asthma cases (“yes” to “bronchial asthma, alone” or “allergy and asthma, together” at Exam 1 or “asthma, onset before age 16,” “asthma, onset age 16 or after,” or “asthma, age of onset unknown” at Exam 5). Individuals with a response of “yes” to the question “lifetime history of chronic pulmonary disease” at Exam 2 were excluded from both cases and controls. All remaining individuals were coded as asthma controls. For the Offspring Cohort, data from Exams 2 and 7 were used. Individuals who answered “yes” to any asthma question were coded as asthma cases (“yes” to “asthma” at Exam 2 or “yes, new” or “yes, old” to “have you had asthma in the interim?” at Exam 7). Individuals with a response of “yes” to “chronic obstructive lung disease” or “chronic bronchitis” at Exam 2 were excluded from both cases and controls. Individuals with a response of “yes, now” or “yes, not now” to “non-cardiovascular medications: bronchodilators and aerosols” were excluded from the controls. All remaining individuals were coded as asthma controls. For the Third Generation Cohort, individuals who answered “yes” to “asthma: was it diagnosed by a doctor or other health professional?” were coded as asthma cases. Individuals who answered “no” to both “asthma: was it diagnosed by a doctor or other health professional?” and “have you ever had asthma?” were coded as asthma controls. Individuals who answered “yes” to any of the following questions were excluded from both the cases and controls: “have you ever had COPD?,” “have you ever had pulmonary fibrosis?,” “have you ever had chronic bronchitis?,” “have you ever had emphysema?.”

*Smoking status:* For the Original Cohort, data from Exam 5 were used. Current smokers answered “smokes now” to the question “history of smoking, present habit.” Former smokers answered “does not smoke now, but smoked formerly,” and never smokers answered “never smoked.” For the Offspring Cohort, data from Exams 1, 2, and 7 were used. Current smokers answered “yes” to “smoked cigarettes regularly in last year” (Exam 7) or “smoking now” (Exam 2). Former smokers answered “no” to “smoked cigarettes regularly in last year” (Exam 7) and “yes” to “ever smoked regularly” (Exam 2) or “smoked at least 1 year” (Exam 1). Never smokers answered “no” to “ever smoked” or “ever smoked regularly” (Exam 2). For the Third Generation Cohort, current smokers answered “yes” to “if ever smoked cigarettes regularly: do you now smoke?.” Former smokers answered “yes” to “have you ever smoked cigarettes regularly?” and “no” to “if ever smoked cigarettes regularly: do you now smoke?.” Never smokers answered “no” to “have you ever smoked cigarettes regularly?.”

*Allergy status:* For the Original Cohort, data from Exam 1 were used. Individuals who answered “allergy, alone” or “allergy and asthma, together” to “history of allergy or asthma” were classified as allergy cases. For the Offspring Cohort, no allergy data were available. For the Third Generation Cohort, individuals who answered “yes” to “have you ever had hay fever (allergy involving the nose and/or eyes)?” were classified as allergy cases.

**Health 2000 Study**

*Description:* The study was conducted in 2000 (E[7](#_ENREF_7)) and included home interview, several questionnaires, laboratory and anthropometrical measurements, spirometry with bronchodilator test and clinical examination of a physician. The data were completed by record linkage with the National Hospital Discharge Register and the National Social Insurance Institutions register data on reimbursement of asthma medication.

*Funding:* The study was mainly funded by the Finnish National Institute for Health and Welfare. H2000 participants were genotyped as part of the HDL extremes study (E[8](#_ENREF_8)) and the Sanger Institute/Wellcome Trust efforts for GENMETS (Genetic Background and Molecular Pathogenesis of Metabolic Syndrome and Its component Risk Factor Traits).

*Asthma status:* **Information about asthma was based on the following question: *“Has a doctor ever diagnosed you with one of the following illnesses?”* One of the listed illnesses was asthma and those who responded “YES” were considered to have asthma. The rest of the participants were taken as controls if their age was less than or equal to 70 years and they never had had chronic bronchitis.**

*Smoking status:*Information about smoking was taken from a question with five alternatives: (1) I smoke daily; (2) I smoke occasionally; (3) I have quit smoking 1-12 months ago; (4) I have quit smoking more than a year ago; (5) I have never smoked. Participants choosing alternatives 1 or 2 were considered as current smokers; those choosing 3 or 4 were considered as ex-smokers; and those choosing 5 were considered as never smokers.

*Allergy status:* **Information about allergy was based on responses under the subheading: *“Other diseases diagnosed by a doctor”.* One of the alternatives gives was *“allergy, rhinitis; for example hay fever or other allergic rhinitis”* Those responding “YES” to this item were considered as having allergy.**

**Helsinki Birth Cohort Study (HBCS)**

*Description:* The study includes 8760 subjects born in Helsinki between 1934 and 1944. Between 2001 and 2004, a representative subset of 928 males and 1075 females participated in a clinical study focusing upon cardiovascular and metabolic outcomes and cognitive functions (E[9](#_ENREF_9)). Information on asthma, smoking and alcohol intake is available from questionnaires for 2003 individuals who participated in the clinical study. Information on hospitalization due to alcohol abuse is available from the National Hospital Discharge Register. Psychological questionnaires have been used to assess personality characteristics including data on impulsivity. GWAS has been done on these individuals participating in the clinical study (n~2000) at the mean age of ~62 years.

*Funding:* Financial support was received from the Academy of Finland, Samfundet Folkhälsan, Finnish Diabetes Research Foundation, Finska Läkaresällskapet, Finnish Foundation for Cardiovascular Research; Yrjö Jahnsson Foundation, Foundation Liv och Hälsa and Academy of Finland (grants number 129287 and134839).

*Asthma status:* Information about asthma was based on the following question: *“Have you ever had any of the following illnesses diagnosed or treated by a doctor?”* One of the listed illnesses was *“Asthma of the lungs”* and those responding “YES” to this item were considered as having asthma. Those participants who responded to the same question as having emphysema or chronic bronchitis were excluded and the others were taken as controls. All participants were less than 70 years of age.

*Smoking status:* Information about smoking was taken from a question with three alternatives: (1) I smoke daily; (2) I smoke occasionally; (3) I have never smoked. Participants choosing alternatives 1 were considered as current smokers; those choosing 2 were considered as ex-smokers; and those choosing 3 were considered as never smokers.

*Allergy status:* Information about allergy was based on the following question: *“Do you have following allergic symptoms? Nasal allergy, hay fever, stuffy nose”* Participants responding “YES” were considered as having allergy.

**Northern Finland Birth Cohort of 1966 (NFBC1966)**

*Description:* This is a prospective follow-up study of 12,058 live births from the two northernmost provinces of Finland, Oulu and Lapland, and cover 96% of the children born in that region between January 1 and December 31, 1966. In 1997, at age 30 years, 8463 survivors still living in Northern Finland or in the capital area received a postal questionnaire and invitation to clinical examinations including DNA sample (71% participated) (E[10](#_ENREF_10), [11](#_ENREF_11)). Please see <http://kelo.oulu.fi/NFBC/> for more details about the study. Informed consent for the use of the data including DNA was obtained from all subjects. The study was approved by the ethics committees in Oulu (Finland) and Oxford (UK) universities in accordance with the Declaration of Helsinki.

*Funding:* Financial support from the Academy of Finland (project grants 104781, 1114194, 120315 and Center of Excellence in Complex Disease Genetics), Oulu University Hospital, Biocenter Oulu, University of Oulu, Finland, the European Commission (EURO-BLCS, Framework 5 award QLG1-CT-2000-01643), NHLBI grant 5R01HL087679-02 through the STAMPEED program (1RL1MH083268-01), ENGAGE project and grant agreement HEALTH-F4-2007-201413, and the Medical Research Council (studentship grant G0500539). We thank Professor Paula Rantakallio (launch of NFBC1966 and 1986), Ms Outi Tornwall and Ms Minttu Jussila (DNA biobanking). DNA extractions, sample quality controls, biobank up-keeping and aliquotting was performed in the National Institute for Health and Welfare, Biomedicum Helsinki, Finland and supported financially by the Academy of Finland and Biocentrum Helsinki.

*Asthma status:* We defined asthmatic cases as those who answered yes to BOTH conditions which are parts of Question 31 of the main questionnaire administered in 1997 when participants were aged 31 years: 1) “*have you ever asthma during the last 12 months or more than a year ago?”* and 2) “*has this been verified or treated by doctor?”* The remaining individuals served as healthy controls if they did not have any of the following conditions: self-reported asthma but not verified or treated by doctor diagnosis (n=196); individuals with FEV1 less than 70% of predicted for sex and height (n=19); individuals who reported they ever had emphysema, chronic bronchitis, pulmonary bronchial (n=211) or doctor-diagnosed chronic cough (n=402).

*Smoking status:* First we defined the never/ever smoking status by Question 79 *“Have you ever smoked in your life?”* of 31-year questionnaire. Among the Ever smokers, we distinguish the Current Smokers if they answered said that they smoke on at least one day per week to Question 82 *“Do you smoke nowadays?”*. We excluded 43 ever smokers whom we could not distinguish if they were current or ex-smokers.

*Allergy status:* We defined an individual as allergic if they answered yes for either having hay fever or allergic rhinitis during the last 12 months or more than a year ago in Question 31 of main questionnaire.

**Young Finns Study (YFS)**

*Description:* This is a longitudinal population study sample on the evolution of cardiovascular risk factors from childhood to adulthood (E[12](#_ENREF_12)). The first cross-sectional survey was conducted in 1980 in five Finnish university cities and included 3,596 participants who were in the age groups of 3, 6, 9, 12, 15, and 18 years and were randomly chosen from the national population register; equal ratios of males and females were selected in each age group. In 2007, 2204 subjects now aged 30 to 45 years participated in the latest follow-up study.

*Funding:* Financially supported by the Academy of Finland (grant numbers 126925, 121584, and 124282); the Social Insurance Institution of Finland; Kuopio, Tampere, and Turku University Hospital Medical Funds; the Juho Vainio Foundation; the Paavo Nurmi Foundation; the Tampere Tuberculosis Foundation; the Finnish Foundation of Cardiovascular Research; and the Finnish Cultural Foundation.

*Asthma status:* Information about asthma was based on a following question: *“Do you have at the moment or have you had a long-term illness, handicap or injury diagnosed by a doctor?”* Those responding “YES” and specifying among the given alternatives “Asthma of the lungs”were considered as having asthma. The rest of the participants were taken as controls, except those who reported having chronic bronchitis. All YFS participants were less than 70 years of age.

*Smoking status:* Information about smoking was based on a question with 5 response alternatives as follows: (1) I smoke once a day or more often than once a day; (2) I smoke once a week or more often than once a week but not daily; (3) I smoke less often than once a week; (4) I have stopped smoking; (5) I have never smoked. Participants choosing any of the alternatives 1-3 were taken as current smokers; those choosing the alternative 4 were taken as ex-smokers and those choosing the alternative 5 were taken as never smokers.

*Allergy status:* Information about allergy was based on the same question as above, i.e., *“Do you have at the moment or have you had a long-term illness, handicap or injury diagnosed by a doctor?”*Those responding “YES”and specifying among the given alternatives *“Allergic rhinitis, for example hay fever”*were taken as having allergy.

**Stage 2 – Brief study description, funding acknowledgement and phenotype definition**

**1958 British Birth Cohort (B58C)**

*Description:* This is a nationwide British birth cohort including participants born in a particular week of 1958. Details of the B58C biomedical follow-up at age 44.5 have been previously reported and a full technical report is available online (<http://www.b58cgene.sgul.ac.uk/report.php>).

*Funding:* The phenotype and genotype data are from the British 1958 Birth Cohort DNA collection, funded by the Medical Research Council grant G0000934 and the Wellcome Trust grant 068545/Z/02. Genotyping for the B58C-WTCCC subset was funded by the Wellcome Trust grant 076113/B/04/Z (E[13](#_ENREF_13)). The B58C-T1DGC genotyping utilized resources provided by the Type 1 Diabetes Genetics Consortium (E[14](#_ENREF_14)), a collaborative clinical study sponsored by the National Institute of Diabetes and Digestive and Kidney Diseases (NIDDK), National Institute of Allergy and Infectious Diseases (NIAID), National Human Genome Research Institute (NHGRI), National Institute of Child Health and Human Development (NICHD), and Juvenile Diabetes Research Foundation International (JDRF) and supported by U01 DK062418. B58C-T1DGC GWAS data were deposited by the Diabetes and Inflammation Laboratory, Cambridge Institute for Medical Research (CIMR), University of Cambridge, which is funded by Juvenile Diabetes Research Foundation International, the Wellcome Trust and the National Institute for Health Research Cambridge Biomedical Research Centre; the CIMR is in receipt of a Wellcome Trust Strategic Award (079895). Genotyping for the B58C-GABRIEL and for ECRHS was supported by a contract from the European Commission as part of GABRIEL (A multidisciplinary study to identify the genetic and environmental causes of asthma in the European Community) contract number 018996 under the Integrated Program LSH-2004-1.2.5-1 Post genomic approaches to understand the molecular basis of asthma aiming at a preventive or therapeutic control and grants from the French Ministry of Research (E[15](#_ENREF_15)).

*Asthma status:* Asthma is defined as self-reported ever at either age 33 or age 42 via administered interviews. Healthy controls exclude any history of asthma, wheezing or wheezing bronchitis as reported in the interviews at age 7, 11, 16 and 23, plus exclusions for bronchitis at age 42 and FEV1 less than 70% predicted for sex and height at age 42.

**Australian asthma genetics consortium (AAGC)**

We carried out a GWAS in 2,110 physician-diagnosed asthmatics and 3,857 controls of European ancestry from Australia. Participants were drawn from two cohorts that are described in detail elsewhere (Ferreira et al., in press): the Australian Asthma Genetics Consortium (AAGC) cohort (n=1,810) and the Queensland Institute of Medical Research (QIMR) GWAS cohort (n=4,157). Amongst the 2,110 asthmatic cases, 759 (36%) were diagnosed through clinical examination and 1,351 (64%) reported a lifetime doctor diagnosis of asthma in epidemiological questionnaires. With respect to disease onset, 1,269 (60%) subjects were classified has having childhood asthma (defined by an age-of-onset at or before age 16), 515 (24%) subjects with later onset asthma (age-of-onset after the age of 16) and 326 (16%) with unknown age-of-onset. Fifty-eight percent of asthmatics were atopic, as defined by a positive skin prick test (SPT) response to at least one common allergen; 68% had at least one first-degree relative with asthma; and 36% reported lifetime smoking.

The 3,857 controls included 2,030 (52.6%) individuals who were classified as asthma-free based on clinical examination (3.0%) or epidemiological questionnaires (49.6%). The remaining 1,827 (47.4%) individuals provided no information about their asthma status and were included in the analysis as controls to improve power. SPT information and lifetime smoking status was unavailable for most controls. Overall, the mean age of participants was 34 years (sd=16.2, range 2 to 92) and 45.2% were males. This dataset does not include 1,230 samples from the Busselton cohort analysed in the GABRIEL study.

***Australian Asthma Genetics Consortium collaborators:*** Svetlana Baltic^1^, Mark Jenkins^2^, Dale R. Nyholt^3^, Catherine Hayden^4^, Patrick Danoy^5^, Faang Cheah^1^, John L. Hopper^2^, Stephen R. Leeder^6^, Haydn Walters^7^, Graham Jones^8^, Désirée Mészáros^9^, Mary Roberts^10^, Melissa C. Southey^11^, Euan R. Tovey^12^, Loren Price^1^, Margaret J. Wright^3^, Scott D. Gordon^3^, Li P. Chung^1^, Anjali K. Henders^3^, Graham Giles^13^, Paul S. Thomas^14^, Suzanna Temple^1^, John B. Whitfield^3^, Ian Feather^15^, Stephen Morrison^16^, Chalermchai Mitrpant^1^, Warwick J. Britton^17^, David John^2^, Andrew S. Kemp^18^

^1^ Lung Institute of WA and Centre for Asthma, Allergy and Respiratory Research, University of WA, Perth, Australia.

^2^ Centre for Molecular, Environmental, Genetic and Analytic Epidemiology, University of Melbourne, Melbourne, Australia.

^3^ The Queensland Institute of Medical Research, Brisbane, Australia.

^4^ School of Paediatrics and Child Health, Princess Margaret Hospital for Children, Perth, Australia.

^5^ University of Queensland Diamantina Institute, Princess Alexandra Hospital, Brisbane, Australia.

^6^ Australian Health Policy Institute, University of Sydney, Sydney, Australia

^7^ Menzies Research Institute, Hobart, Australia.

^8^ University of Western Sydney, Penrith, Australia

^9^ Menzies Research Institute, Hobart, Australia.

^10^ Department of Respiratory Medicine, Royal Children's Hospital, Parkville, Australia.

^11^ Department of Pathology, The University of Melbourne, Melbourne, Australia.

^12^ Woolcock Institute of Medical Research, University of Sydney, Sydney, Australia.

^13^ Cancer Epidemiology Centre, The Cancer Council Victoria, Melbourne, Australia.

^14^ Faculty of Medicine, University of New South Wales, Sydney, Australia.

^15^ Gold Coast Hospital, Southport, Australia.

^16^ University of Queensland, Brisbane, Australia.
^17^ Centenary Institute and University of Sydney, Camperdown, Australia.

^18^ The Children's Hospital, Westmead, Sydney, Australia.

**European Community Respiratory Health Survey (ECRHS)**

*Description:* This is a multicentre, mainly European, population based survey carried out in 1990s (E[16](#_ENREF_16)) with a follow-up starting in 1998 (E[17](#_ENREF_17)). Questionnaire information is based on information collected through interviewer administered questionnaire (forms available at <http://www.ecrhs.org/>). Participants were aged 20 – 48 (mean of 34) at the first survey and 28 – 56 (mean age 43) at the second survey. Samples were genotyped as part of the GABRIEL consortium (E[15](#_ENREF_15)) and were collected at the follow-up and were mainly European participants and included an enrichment of asthma cases.

*Funding:* The co-ordination of follow-up of was supported by the European Commission, as part of their Quality of Life programme. The following bodies funded the local studies in ECRHS II: **Albacete:** Fondo de Investigaciones Santarias (FIS) (grant code: 97/0035-01, 99/0034-01 and 99/0034-02), Hospital Universitario de Albacete, Consejeria de Sanidad; **Barcelona**: SEPAR, Public Health Service (grant code: R01 HL62633-01), Fondo de Investigaciones Santarias (FIS) (grant code: 97/0035-01, 99/0034-01 and 99/0034-02) CIRIT (grant code: 1999SGR 00241) Red Respira ISCII; CIBER Epidemiologia y Salud Pública (CIBERESP), Spain **Basel**: Swiss National Science Foundation, Swiss Federal Office for Education & Science, Swiss National Accident Insurance Fund (SUVA), USC NIEHS Center grant 5P30 ES07048; **Bergen**: Norwegian Research Council, Norwegian Asthma & Allergy Association (NAAF), Glaxo Wellcome AS, Norway Research Fund; **Erfurt**: GSF-National Research Centre for Environment & Health, Deutsche Forschungsgemeinschaft (DFG) (grant code FR 1526/1-1); **Galdakao**: Basque Health Dept; **Grenoble**: Programme Hospitalier de Recherche Clinique-DRC de Grenoble 2000 no. 2610, Ministry of Health, Direction de la Recherche Clinique, CHU de Grenoble, Ministere de l'Emploi et de la Solidarite, Direction Generale de la Sante, Comite des Maladies Respiratoires de l’Isere; **Hamburg:** GSF-National Reasearch Centre for Environment & Health, Deutsche Forschungsgemeinschaft (DFG) (grant code MA 711/4-1); **Ipswich and Norwich**: Asthma UK (formerly known as National Asthma Campaign); **Huelva**: Fondo de Investigaciones Santarias (FIS) (grant code: 97/0035-01, 99/0034-01 and 99/0034-02); **Oviedo**: Fondo de Investigaciones Santarias (FIS) (grant code: 97/0035-01, 99/0034-01 and 99/0034-02) ; **Paris**: Ministere de l'Emploi et de la Solidarite, Direction Generale de la Sante, UCB-Pharma (France), Aventis (France), Glaxo France, Programme Hospitalier de Recherche Clinique-DRC de Grenoble 2000 no. 2610, Ministry of Health, Direction de la Recherche Clinique, CHU de Grenoble; **Tartu**: Estonian Science Foundation; **Umeå**: Swedish Heart Lung Foundation, Swedish Foundation for Health Care Sciences & Allergy Research, Swedish Asthma & Allergy Foundation, Swedish Cancer & Allergy Foundation; **Uppsala**: Swedish Heart Lung Foundation, Swedish Foundation for Health Care Sciences & Allergy Research, Swedish Asthma & Allergy Foundation, Swedish Cancer & Allergy Foundation; *Financial support for ECRHS I for centres in ECRHS II was provided by*: Ministère de la Santé, Glaxo France, Insitut Pneumologique d'Aquitaine, Contrat de Plan Etat-Région Languedoc-Rousillon, CNMATS, CNMRT (90MR/10, 91AF/6), Ministre delegué de la santé, RNSP, France; GSF, and the Bundesminister für Forschung und Technologie, Bonn, Germany; Norwegian Research Council project no. 101422/310; Ministero Sanidad y Consumo FIS (grants #91/0016060/00E-05E and #93/0393), and grants from Hospital General de Albacete, Hospital General Juan Ramón Jiménenz, Consejeria de Sanidad Principado de Asturias, Spain; The Swedish Medical Research Council, the Swedish Heart Lung Foundation, the Swedish Association against Asthma and Allergy; Swiss National Science Foundation grant 4026-28099; National Asthma Campaign, British Lung Foundation, Department of Health, South Thames Regional Health Authority, UK.

*Asthma status:* We define asthmatic cases if they responded positively to BOTH of the following questions: 1) ever had asthma at first survey (Question 13) or second survey (Question 14) and 2) if it was verified by doctor diagnosis in second survey (Question 14 part 1). The remaining individuals served as healthy controls if they did not have any of the following conditions: self-reported asthma without doctor diagnosis (n=36); reported ever having wheeze in second survey (n=285); FEV1 less than 70% predicted for age, sex and height (n=10).

**European Prospective Investigation of Cancer, Norfolk (EPIC-Norfolk)**

*Description:* EPIC-Norfolk is part of the large multi-centre Europe-wide EPIC programme looking at the connection between diet and cancer (E[18](#_ENREF_18)). There are over 30,000 participants aged 45 – 70 at recruitment who lived in Norwich and the surrounding towns and rural areas. They have been contributing information about their diet, lifestyle and health through questionnaires, and through health checks carried out by EPIC nurses. In 2006, a GWAS for obesity was carried out on 3,867 individuals (E[19](#_ENREF_19)). A case-cohort design was used in which the subcohort (*N=*2,566) was a random sample of the cohort at baseline and cases were part of the remaining individuals with a value of BMI being 30 kg/m^2^ or greater (N*=*1,301). Unlike controls in the commonly used case-control design, the subcohort was an unselected population sample allowing for a variety of traits to be investigated.

*Funding:* Supported by research programme grant funding from the Cancer Research Campaign and the Medical Research Council, with additional support from Stroke Association, British Heart Foundation, Department of Health, Europe Against Cancer Programme Commission of the European Union, the Food Standards Agency and the Wellcome Trust.

*Asthma status:* Asthmatics were defined as a positive response to the question *"Has the doctor ever told you that you have asthma?"* which was asked at baseline survey. The remaining individuals served as healthy controls unless they had bronchitis or FEV1 < 70% predicted for age, height and sex.

**REFERENCES**

E1. Vartiainen E, Laatikainen T, Peltonen M, Juolevi A, Mannisto S, Sundvall J, Jousilahti P, Salomaa V, Valsta L, Puska P**.** Thirty-five-year trends in cardiovascular risk factors in Finland. *Int J Epidemiol*. 2010;39(2):504-18.

E2. Ripatti S, Tikkanen E, Orho-Melander M, Havulinna AS, Silander K, Sharma A, Guiducci C, Perola M, Jula A, Sinisalo J, Lokki ML, Nieminen MS, Melander O, Salomaa V, Peltonen L, Kathiresan S**.** A multilocus genetic risk score for coronary heart disease: case-control and prospective cohort analyses. *Lancet*. 2010;376(9750):1393-400.

E3. Kathiresan S, Voight BF, Purcell S, Musunuru K, Ardissino D, Mannucci PM, Anand S, Engert JC, Samani NJ, Schunkert H, Erdmann J, Reilly MP, Rader DJ, Morgan T, Spertus JA, Stoll M, Girelli D, McKeown PP, Patterson CC, Siscovick DS, O'Donnell CJ, Elosua R, Peltonen L, Salomaa V, Schwartz SM, Melander O, Altshuler D, Merlini PA, Berzuini C, Bernardinelli L, Peyvandi F, Tubaro M, Celli P, Ferrario M, Fetiveau R, Marziliano N, Casari G, Galli M, Ribichini F, Rossi M, Bernardi F, Zonzin P, Piazza A, Yee J, Friedlander Y, Marrugat J, Lucas G, Subirana I, Sala J, Ramos R, Meigs JB, Williams G, Nathan DM, MacRae CA, Havulinna AS, Berglund G, Hirschhorn JN, Asselta R, Duga S, Spreafico M, Daly MJ, Nemesh J, Korn JM, McCarroll SA, Surti A, Guiducci C, Gianniny L, Mirel D, Parkin M, Burtt N, Gabriel SB, Thompson JR, Braund PS, Wright BJ, Balmforth AJ, Ball SG, Hall AS, Linsel-Nitschke P, Lieb W, Ziegler A, Konig I, Hengstenberg C, Fischer M, Stark K, Grosshennig A, Preuss M, Wichmann HE, Schreiber S, Ouwehand W, Deloukas P, Scholz M, Cambien F, Li M, Chen Z, Wilensky R, Matthai W, Qasim A, Hakonarson HH, Devaney J, Burnett MS, Pichard AD, Kent KM, Satler L, Lindsay JM, Waksman R, Knouff CW, Waterworth DM, Walker MC, Mooser V, Epstein SE, Scheffold T, Berger K, Huge A, Martinelli N, Olivieri O, Corrocher R, McKeown P, Erdmann E, Konig IR, Holm H, Thorleifsson G, Thorsteinsdottir U, Stefansson K, Do R, Xie C, Siscovick D**.** Genome-wide association of early-onset myocardial infarction with single nucleotide polymorphisms and copy number variants. *Nat Genet*. 2009;41(3):334-41.

E4. Dawber TR, Meadors GF, Moore FE**.** Epidemiologic approaches to heart disease: the Framingham study. *Am J Public Health*. 1951;41:179-286.

E5. Feinleib M, Kannel WB, Garrison RJ, McNamara PM, Castelli WP**.** The Framingham Offspring Study. Design and preliminary data. *Prev Med*. 1975;4(4):518-25.

E6. Splansky GL, Corey D, Yang Q, Atwood LD, Cupples LA, Benjamin EJ, D'Agostino RB, Sr., Fox CS, Larson MG, Murabito JM, O'Donnell CJ, Vasan RS, Wolf PA, Levy D**.** The Third Generation Cohort of the National Heart, Lung, and Blood Institute's Framingham Heart Study: design, recruitment, and initial examination. *Am J Epidemiol*. 2007;165(11):1328-35.

E7. Aromaa A, Koskinen S, eds. Health and functional capacity in Finland. Baseline Results of the Health 2000 Health Examination Survey. Helsinki: National Public Health Institute KTL; 2004.

E8. Perttila J, Merikanto K, Naukkarinen J, Surakka I, Martin NW, Tanhuanpaa K, Grimard V, Taskinen MR, Thiele C, Salomaa V, Jula A, Perola M, Virtanen I, Peltonen L, Olkkonen VM**.** OSBPL10, a novel candidate gene for high triglyceride trait in dyslipidemic Finnish subjects, regulates cellular lipid metabolism. *J Mol Med (Berl)*. 2009;87(8):825-35.

E9. Barker DJ, Osmond C, Forsen TJ, Kajantie E, Eriksson JG**.** Trajectories of growth among children who have coronary events as adults. *N Engl J Med*. 2005;353(17):1802-9.

E10. Jarvelin MR, Sovio U, King V, Lauren L, Xu B, McCarthy MI, Hartikainen AL, Laitinen J, Zitting P, Rantakallio P, Elliott P**.** Early life factors and blood pressure at age 31 years in the 1966 northern Finland birth cohort. *Hypertension*. 2004;44(6):838-46.

E11. Canoy D, Pekkanen J, Elliott P, Pouta A, Laitinen J, Hartikainen AL, Zitting P, Patel S, Little MP, Jarvelin MR**.** Early growth and adult respiratory function in men and women followed from the fetal period to adulthood. *Thorax*. 2007;62(5):396-402.

E12. Raitakari OT, Juonala M, Ronnemaa T, Keltikangas-Jarvinen L, Rasanen L, Pietikainen M, Hutri-Kahonen N, Taittonen L, Jokinen E, Marniemi J, Jula A, Telama R, Kahonen M, Lehtimaki T, Akerblom HK, Viikari JS**.** Cohort profile: the cardiovascular risk in Young Finns Study. *Int J Epidemiol*. 2008;37(6):1220-6.

E13. Craddock N, Hurles ME, Cardin N, Pearson RD, Plagnol V, Robson S, Vukcevic D, Barnes C, Conrad DF, Giannoulatou E, Holmes C, Marchini JL, Stirrups K, Tobin MD, Wain LV, Yau C, Aerts J, Ahmad T, Andrews TD, Arbury H, Attwood A, Auton A, Ball SG, Balmforth AJ, Barrett JC, Barroso I, Barton A, Bennett AJ, Bhaskar S, Blaszczyk K, Bowes J, Brand OJ, Braund PS, Bredin F, Breen G, Brown MJ, Bruce IN, Bull J, Burren OS, Burton J, Byrnes J, Caesar S, Clee CM, Coffey AJ, Connell JM, Cooper JD, Dominiczak AF, Downes K, Drummond HE, Dudakia D, Dunham A, Ebbs B, Eccles D, Edkins S, Edwards C, Elliot A, Emery P, Evans DM, Evans G, Eyre S, Farmer A, Ferrier IN, Feuk L, Fitzgerald T, Flynn E, Forbes A, Forty L, Franklyn JA, Freathy RM, Gibbs P, Gilbert P, Gokumen O, Gordon-Smith K, Gray E, Green E, Groves CJ, Grozeva D, Gwilliam R, Hall A, Hammond N, Hardy M, Harrison P, Hassanali N, Hebaishi H, Hines S, Hinks A, Hitman GA, Hocking L, Howard E, Howard P, Howson JM, Hughes D, Hunt S, Isaacs JD, Jain M, Jewell DP, Johnson T, Jolley JD, Jones IR, Jones LA, Kirov G, Langford CF, Lango-Allen H, Lathrop GM, Lee J, Lee KL, Lees C, Lewis K, Lindgren CM, Maisuria-Armer M, Maller J, Mansfield J, Martin P, Massey DC, McArdle WL, McGuffin P, McLay KE, Mentzer A, Mimmack ML, Morgan AE, Morris AP, Mowat C, Myers S, Newman W, Nimmo ER, O'Donovan MC, Onipinla A, Onyiah I, Ovington NR, Owen MJ, Palin K, Parnell K, Pernet D, Perry JR, Phillips A, Pinto D, Prescott NJ, Prokopenko I, Quail MA, Rafelt S, Rayner NW, Redon R, Reid DM, Renwick, Ring SM, Robertson N, Russell E, St Clair D, Sambrook JG, Sanderson JD, Schuilenburg H, Scott CE, Scott R, Seal S, Shaw-Hawkins S, Shields BM, Simmonds MJ, Smyth DJ, Somaskantharajah E, Spanova K, Steer S, Stephens J, Stevens HE, Stone MA, Su Z, Symmons DP, Thompson JR, Thomson W, Travers ME, Turnbull C, Valsesia A, Walker M, Walker NM, Wallace C, Warren-Perry M, Watkins NA, Webster J, Weedon MN, Wilson AG, Woodburn M, Wordsworth BP, Young AH, Zeggini E, Carter NP, Frayling TM, Lee C, McVean G, Munroe PB, Palotie A, Sawcer SJ, Scherer SW, Strachan DP, Tyler-Smith C, Brown MA, Burton PR, Caulfield MJ, Compston A, Farrall M, Gough SC, Hall AS, Hattersley AT, Hill AV, Mathew CG, Pembrey M, Satsangi J, Stratton MR, Worthington J, Deloukas P, Duncanson A, Kwiatkowski DP, McCarthy MI, Ouwehand W, Parkes M, Rahman N, Todd JA, Samani NJ, Donnelly P**.** Genome-wide association study of CNVs in 16,000 cases of eight common diseases and 3,000 shared controls. *Nature*. 2010;464(7289):713-20.

E14. Barrett JC, Clayton DG, Concannon P, Akolkar B, Cooper JD, Erlich HA, Julier C, Morahan G, Nerup J, Nierras C, Plagnol V, Pociot F, Schuilenburg H, Smyth DJ, Stevens H, Todd JA, Walker NM, Rich SS**.** Genome-wide association study and meta-analysis find that over 40 loci affect risk of type 1 diabetes. *Nat Genet*. 2009;41(6):703-7.

E15. Moffatt MF, Gut IG, Demenais F, Strachan DP, Bouzigon E, Heath S, von Mutius E, Farrall M, Lathrop M, Cookson WO**.** A large-scale, consortium-based genomewide association study of asthma. *N Engl J Med*. 2010;363(13):1211-21.

E16. Burney PG, Luczynska C, Chinn S, Jarvis D**.** The European Community Respiratory Health Survey. *Eur Respir J*. 1994;7(5):954-60.

E17. The European Community Respiratory Health Survey II. *Eur Respir J*. 2002;20(5):1071-9.

E18. Day N, Oakes S, Luben R, Khaw KT, Bingham S, Welch A, Wareham N**.** EPIC-Norfolk: study design and characteristics of the cohort. European Prospective Investigation of Cancer. *Br J Cancer*. 1999;80 Suppl 1:95-103.

E19. Loos RJ, Lindgren CM, Li S, Wheeler E, Zhao JH, Prokopenko I, Inouye M, Freathy RM, Attwood AP, Beckmann JS, Berndt SI, Jacobs KB, Chanock SJ, Hayes RB, Bergmann S, Bennett AJ, Bingham SA, Bochud M, Brown M, Cauchi S, Connell JM, Cooper C, Smith GD, Day I, Dina C, De S, Dermitzakis ET, Doney AS, Elliott KS, Elliott P, Evans DM, Sadaf Farooqi I, Froguel P, Ghori J, Groves CJ, Gwilliam R, Hadley D, Hall AS, Hattersley AT, Hebebrand J, Heid IM, Lamina C, Gieger C, Illig T, Meitinger T, Wichmann HE, Herrera B, Hinney A, Hunt SE, Jarvelin MR, Johnson T, Jolley JD, Karpe F, Keniry A, Khaw KT, Luben RN, Mangino M, Marchini J, McArdle WL, McGinnis R, Meyre D, Munroe PB, Morris AD, Ness AR, Neville MJ, Nica AC, Ong KK, O'Rahilly S, Owen KR, Palmer CN, Papadakis K, Potter S, Pouta A, Qi L, Randall JC, Rayner NW, Ring SM, Sandhu MS, Scherag A, Sims MA, Song K, Soranzo N, Speliotes EK, Syddall HE, Teichmann SA, Timpson NJ, Tobias JH, Uda M, Vogel CI, Wallace C, Waterworth DM, Weedon MN, Willer CJ, Wraight, Yuan X, Zeggini E, Hirschhorn JN, Strachan DP, Ouwehand WH, Caulfield MJ, Samani NJ, Frayling TM, Vollenweider P, Waeber G, Mooser V, Deloukas P, McCarthy MI, Wareham NJ, Barroso I, Kraft P, Hankinson SE, Hunter DJ, Hu FB, Lyon HN, Voight BF, Ridderstrale M, Groop L, Scheet P, Sanna S, Abecasis GR, Albai G, Nagaraja R, Schlessinger D, Jackson AU, Tuomilehto J, Collins FS, Boehnke M, Mohlke KL**.** Common variants near MC4R are associated with fat mass, weight and risk of obesity. *Nat Genet*. 2008;40(6):768-75.

E20. Hirota T, Takahashi A, Kubo M, Tsunoda T, Tomita K, Doi S, Fujita K, Miyatake A, Enomoto T, Miyagawa T, Adachi M, Tanaka H, Niimi A, Matsumoto H, Ito I, Masuko H, Sakamoto T, Hizawa N, Taniguchi M, Lima JJ, Irvin CG, Peters SP, Himes BE, Litonjua AA, Tantisira KG, Weiss ST, Kamatani N, Nakamura Y, Tamari M**.** Genome-wide association study identifies three new susceptibility loci for adult asthma in the Japanese population. *Nat Genet*. 2011.

E21. Noguchi E, Sakamoto H, Hirota T, Ochiai K, Imoto Y, Sakashita M, Kurosaka F, Akasawa A, Yoshihara S, Kanno N, Yamada Y, Shimojo N, Kohno Y, Suzuki Y, Kang MJ, Kwon JW, Hong SJ, Inoue K, Goto Y, Yamashita F, Asada T, Hirose H, Saito I, Fujieda S, Hizawa N, Sakamoto T, Masuko H, Nakamura Y, Nomura I, Tamari M, Arinami T, Yoshida T, Saito H, Matsumoto K**.** Genome-wide association study identifies HLA-DP as a susceptibility gene for pediatric asthma in Asian populations. *PLoS Genet*. 2011;7(7):e1002170.

E22. Gudbjartsson DF, Bjornsdottir US, Halapi E, Helgadottir A, Sulem P, Jonsdottir GM, Thorleifsson G, Helgadottir H, Steinthorsdottir V, Stefansson H, Williams C, Hui J, Beilby J, Warrington NM, James A, Palmer LJ, Koppelman GH, Heinzmann A, Krueger M, Boezen HM, Wheatley A, Altmuller J, Shin HD, Uh ST, Cheong HS, Jonsdottir B, Gislason D, Park CS, Rasmussen LM, Porsbjerg C, Hansen JW, Backer V, Werge T, Janson C, Jonsson UB, Ng MC, Chan J, So WY, Ma R, Shah SH, Granger CB, Quyyumi AA, Levey AI, Vaccarino V, Reilly MP, Rader DJ, Williams MJ, van Rij AM, Jones GT, Trabetti E, Malerba G, Pignatti PF, Boner A, Pescollderungg L, Girelli D, Olivieri O, Martinelli N, Ludviksson BR, Ludviksdottir D, Eyjolfsson GI, Arnar D, Thorgeirsson G, Deichmann K, Thompson PJ, Wjst M, Hall IP, Postma DS, Gislason T, Gulcher J, Kong A, Jonsdottir I, Thorsteinsdottir U, Stefansson K**.** Sequence variants affecting eosinophil numbers associate with asthma and myocardial infarction. *Nat Genet*. 2009;41(3):342-7.

E23. Himes BE, Hunninghake GM, Baurley JW, Rafaels NM, Sleiman P, Strachan DP, Wilk JB, Willis-Owen SA, Klanderman B, Lasky-Su J, Lazarus R, Murphy AJ, Soto-Quiros ME, Avila L, Beaty T, Mathias RA, Ruczinski I, Barnes KC, Celedon JC, Cookson WO, Gauderman WJ, Gilliland FD, Hakonarson H, Lange C, Moffatt MF, O'Connor GT, Raby BA, Silverman EK, Weiss ST**.** Genome-wide association analysis identifies PDE4D as an asthma-susceptibility gene. *Am J Hum Genet*. 2009;84(5):581-93.
