# Supplementary material for: Genome-Wide Association Studies of Asthma in Population-Based Cohorts Confirm Known and Suggested Loci and Identify an Additional Association near HLA
Source: PLoS One. 2012 Sep 28;7(9):e44008. doi: 10.1371/journal.pone.0044008 (PMC3461045; doi:10.1371/journal.pone.0044008)
Supplement: Table S1 — Genotyping platform, calling algorithm, imputation details and software used in Stage 1 and Stage 2 studies. (DOCX) [file pone.0044008.s005.docx]

**Table S1:** Genotyping platform, calling algorithm, imputation details and software used in Stage 1 and Stage 2 studies.

|  |  |  | **QC filters applied before imputation** | | | |  |  |  |  | |  |
| --- | --- | --- | --- | --- | --- | --- | --- | --- | --- | --- | --- | --- |
| **Study name** | **GWAS platform** | **Calling algorithm** | **Subject call rate** | **SNP call rate** | **SNP HWE** | **SNP MAF** | **No of SNPs after filtering** | **Imputation Software** | **HapMap; CEU ver.** | **Genotype-phenotype association software** | | **λ for asthma analysis** |
|  | | | | | | | | | | | | |
| ***Stage 1: GWAs for discovery*** | | | | | | | | | | | | |
| FINRISK – CoroGene | Illumina HumanHap 610 Quad | Beadstudio | 0.95 | 0.95 | 1e-6 | 0.01 | 554,988 | MACH 1.0.16 | 36;22 | PLINK v1.07 | 1.0178 | |
| FINRISK – MIGen | Affymetrix 6.0 GeneChip | Birdseed | 0.95 | 0.95 | 1e-6 | 0.01 | 727,478 | MACH 1.0.16 | 36;22 |  |  |  |
| Health 2000 – GeneMets | Illumina HumanHap 610 Quad | Illuminus | 0.95 | 0.95 | 1e-6 | 0.01 | 555,388 | Mach 1.0.10 | 36;22 |  |  |  |
| Health 2000 – HDL | Illumina 370k | Illuminus | 0.95 | 0.95 | 1e-6 | 0.01 | 339,416 | Mach 1.0.16 | 36;22 |  |  |  |
| Helsinki Birth Cohort | Illumina 670 Quad | Illuminus | 0.95 | 0.95 | 1e-6 | 0.01 | 546,814 | Mach 1.0.16 | 36;22 |  |  |  |
| Young Finns Study | Custom-made Illumina 670k | Illuminus | 0.90 | 0.95 | 1e-6 | 0.01 | 546,677 | MACH 1.0.16 | 36; 22 |  |  |  |
| Framingham Heart Study | Affymetrix 500K | BRLMM | 0.97 | 0.95 | 1.e-6 | 0.01 | 413,905 | MACH 1.0.16 | 36;22 | GEE model in R package GWAF | 1.0146 | |
| Northern Finland Birth Cohort 1966 | Illumina 370K | Beadstudio | none | 0.95 | 1.e-4 | 0.01 | 328,007 | IMPUTE v1.0 | 35; 21 | QUICKTEST v0.94 | 1.0122 | |
|  | | | | | | | | | | | | |
| ***Stage 2: in-silico replication*** | | | | | | | | | | | | |
| Australian Asthma G.C. | Illumina 310K (22%)  Illumina 610K (72%) | Beadstudio | 0.98 | 0.95 | 1e-6 | 0.01 | ~610K | IMPUTE | 1000G | PLINK v1.07 | | NA |
| B58C – WTCCC | Affymetrix 500K | CHIAMO | 0.98 | No | No | No | 490,033 | IMPUTE 0.2.0 | 35;21 | ProbABEL v0.0-7 | | NA |
| B58C - T1DGC | Illumina 550K | ILLUMINUS | 0.98 | No | No | No | 520,010 | MACH 1.0.13 | 35;21 |  |  |  |
| B58C - GABRIEL | Illumina Quad 610 | GenCall | No | 0.97 | 1e-4 | 0.05 | 582,892 | MACH 1.0 | 36; 22 |  |  |  |
| ECRHS | Illumina Quad 610 | GenCall | No | 0.97 | 1e-4 | 0.05 | 582,892 | MACH 1.0 | 36; 22 | ProbABEL v0.0-7 | | NA |
| EPIC obese cases | Affymetrix 500K | BRLMM | 0.94 | 0.90 | 1e-6 | 0.01 | 397,438 | IMPUTE 0.3.1 | 35;21 | SNPTEST 1.1.5 | | NA |
| EPIC population based | Affymetrix 500K | BRLMM | 0.94 | 0.90 | 1e-6 | 0.01 | 397,438 | IMPUTE 0.3.1 | 35;21 | SNPTEST 1.1.5 | | NA |
