# Supplementary material for: Genome-Wide Association Studies of Asthma in Population-Based Cohorts Confirm Known and Suggested Loci and Identify an Additional Association near HLA
Source: PLoS One. 2012 Sep 28;7(9):e44008. doi: 10.1371/journal.pone.0044008 (PMC3461045; doi:10.1371/journal.pone.0044008)
Supplement: Table S2 — Loci with previous genome-wide significant associations to asthma (P<5x10^−8^) in Japanese populations. (DOCX) [file pone.0044008.s006.docx]

**Table S2:** Loci with previous genome-wide significant associations to asthma (P < 5x10^-8^) in Japanese populations

| **Gene region ^a^** | **SNP**^b^ | **Reported** | | | | **APCAT** | | | |
| --- | --- | --- | --- | --- | --- | --- | --- | --- | --- |
|  |  | **Risk Allele** | **Freq** | **OR (95% CI)** | **P value** | **SNPs examined in APCAT ^c^** | **Risk Allele** | **OR_95%_CI** | **P value^d^** |
| ***USP38-GAB1*** | rs7686660 | T | 0.27 | 1.16(1.11–1.21) | 1.87E-12 | rs7686660 | T | 0.97 (0.89,1.05) | 8.00E-01 |
|  |  |  |  |  |  | rs4485768 | G | 0.97 (0.89,1.04) | 8.08E-01 |
|  |  |  |  |  |  | rs10015501 | G | 0.97 (0.89,1.05) | 7.61E-01 |
|  |  |  |  |  |  | rs13126430 | A | 0.98 (0.90,1.05) | 7.47E-01 |
|  |  |  |  |  |  | rs13151714 | G | 0.97 (0.89,1.05) | 7.67E-01 |
| ***HLA* region (*NOTCH4*)** | rs404860 | A | 0.5 | 1.21(1.16–1.25) | 4.07E-23 | rs404860 | A | 0.94 (0.83,1.05) | 8.65E-01 |
| ***HLA* region**  **(*HLA-DPB1*)** | rs987870 | C | 0.17 | 1.33(1.20–1.47) | 2.3E-10 | rs987870 | C | 0.92 (0.79,1.05) | 8.84E-01 |
| ***IKZF4*** | rs1701704 | G | 0.18 | 1.19(1.14–1.25) | 2.33E-13 | rs1701704 | G | 1.13 (1.05,1.21) | 1.32E-03 |
|  |  |  |  |  |  | rs773108 | G | 1.13 (1.05,1.21) | 1.09E-03 |
|  |  |  |  |  |  | rs773114 | T | 1.12 (1.04,1.19) | 2.03E-03 |
|  |  |  |  |  |  | rs2292239 | G | 1.12 (1.04,1.2) | 3.43E-03 |
|  |  |  |  |  |  | rs11171739 | C | 1.10 (1.03,1.18) | 5.83E-03 |
|  |  |  |  |  |  | rs2069408 | G | 1.08 (1.00,1.16) | 2.91E-02 |
| ***GATA3/TAF3*** | rs10508372 | C | 0.43 | 1.16(1.12–1.21) | 1.79E-23 | rs10508372 | C | 1.08 (0.94,1.23) | 1.40E-01 |
|  |  |  |  |  |  | rs10905491 | T | 1.16 (0.93,1.40) | 8.99E-01 |
|  |  |  |  |  |  | rs10905488 | T | 1.08 (0.94,1.23) | 8.60E-01 |

^a^Gene shown is nearest gene to associated SNP. SNPs from the same locus are grouped together and shaded in the same color. ^b^All SNPs are from Hirota et al. (2011) (E[20](#_ENREF_20)) except for rs987870, which is from Noguchi et al. (2011) (E[21](#_ENREF_21)). ^c^All SNPs in LD with r^2^ > 0.8 with the reported SNP in the Hapmap JPT reference panel were collected, and a “clumped” set of SNPs was generated (only one SNP from a pair within 100 kb and pairwise r^2^ > 0.8in HapMap CEU). The association results in APCAT are reported for the most strongly associated SNPs in each clump, for each locus. ^d^APCAT P values are one-tailed with respect to the direction of the risk-increasing allele in the original report.
