# Supplementary material for: Genome-Wide Association Studies of Asthma in Population-Based Cohorts Confirm Known and Suggested Loci and Identify an Additional Association near HLA
Source: PLoS One. 2012 Sep 28;7(9):e44008. doi: 10.1371/journal.pone.0044008 (PMC3461045; doi:10.1371/journal.pone.0044008)
Supplement: Table S3 — Conditional analysis on novel signals in APCAT. (DOCX) [file pone.0044008.s007.docx]

**Table S3:** Conditional analysis on novel signals in APCAT

| **IL1RL1/IL18R1** SNP1 = rs13408661 (APCAT signal) & coded allele is G SNP2 = rs3771166 (GABRIEL signal) (E[15](#_ENREF_15)) & coded allele is G *Distance=31,140 bases and r^2^=0.157* | | | | |
| --- | --- | --- | --- | --- |
|  | ***FINRISK, H2000, HBC, YFS*** | ***FraminghamHS*** | ***NFBC66*** | ***pooled*** |
| **Frequency of coded allele of SNP1** | 83.59% | 83.92% | 83.69% | 83.81% |
| **Frequency of coded allele of SNP2** | 58.97% | 62.67% | 57.87% | 60.02% |
| **SNP1 + covs** | 1.18 (0.99, 1.40); p=6.3e-2 | 1.31 (1.11, 1.54); p=1.5e-3 | 1.49 (1.17, 1.88); p=1.0e-3 | **1.29(1.18,1.4);p=3.84E-06** |
| **SNP2 + covs** | 1.04 (0.91, 1.17); p=5.8e-1 | 1.10 (0.98, 1.24); p=1.1e-1 | 1.29 (1.10, 1.52); p=1.5e-3 | 1.12(1.04,1.19);p=4.7E-03 |
| **SNP1 conditional on SNP2 + covs** | 1.21 (0.99, 1.47); p=6.3e-2 | 1.31 (1.08, 1.59); p=5.7e-3 | 1.33 (1.01, 1.74); p=4.3e-2 | **1.27(1.15,1.4);p=1.23E-04** |
| **SNP2 conditional on SNP1 + covs** | 0.97 (0.83, 1.11); p=6.3e-1 | 0.99 (0.86, 1.14); p=8.8e-1 | 1.17 (0.97, 1.40); p=9.9e-2 | 1.02(0.93,1.11);p=6.9E-01 |
|  |  |  |  |  |
| **IL1RL1/IL18R1** SNP1 = rs13408661 (APCAT signal) & coded allele is G SNP2 = rs1420101(Gudbjartsson et al.) (E[22](#_ENREF_22)) & coded allele is T *Distance=2,634 bases and r^2^=0.053* | | | | |
|  | ***FINRISK, H2000, HBC, YFS*** | ***FraminghamHS*** | ***NFBC66*** | ***pooled*** |
| **Frequency of coded allele of SNP1** | 83.59% | 83.92% | 83.69% | 83.81% |
| **Frequency of coded allele of SNP2** | 31.95% | 39.03% | 31.95% | 34.45% |
| **SNP1 + covs** | 1.18 (0.99, 1.40); p=6.3e-2 | 1.31 (1.11, 1.54); p=1.5e-3 | 1.49 (1.17, 1.88); p=1.0e-3 | **1.29(1.18,1.4);p=3.84E-06** |
| **SNP2 + covs** | 1.13 (1.00, 1.26); p=7.1e-1 | 1.14 (1.02, 1.25); p=3.2e-1 | 1.24 (1.09 1.40); p=6.5e-3 | 1.16(1.08,1.23);p=2.0E-04 |
| **SNP1 conditional on SNP2 + covs** | 1.14 (0.96, 1.32); p=1.6e-1 | 1.27 (1.09, 1.44); p=7.7e-3 | 1.40 (1.15, 1.64); p=7.83e-3 | **1.24(1.13,1.35);p=1.5E-04** |
| **SNP2 conditional on SNP1 + covs** | 1.09 (0.96, 1.23); p=1.9e-1 | 1.08 (0.96, 1.20); p=2.2e-1 | 1.16 (0.99, 1.32); p=7.8e-2 | 1.10(1.02,1.18);p=1.5E-02 |
|  |  |  |  |  |
| ***HLA region*** SNP1= rs9268516 (APCAT signal) & coded allele is T SNP2 = rs9272346 (proxy for GABRIEL rs9273349 with *r*^2^=1) & coded allele is G *Distance=224,883 bases and Rsquared=0.336* | | | | |
|  | ***FINRISK, H2000, HBC, YFS*** | ***FraminghamHS*** | ***NFBC66*** | ***Pooled*** |
| **Frequency of coded allele of SNP1** | 20.76% | 27.25% | 22.46% | 23.92% |
| **Frequency of coded allele of SNP2** | 48.26% | 39.94% | 51.28% | 46.27% |
| **SNP1 + covs** | 1.24 (1.07, 1.43); p=3.6e-3 | 1.30 (1.14, 1.48); p=5.4e-5 | 1.22 (1.01, 1.46); p=3.5e-2 | **1.26(1.17,1.35);p=1.256E-07** |
| **SNP2 + covs** | 0.83 (0.73, 0.94); p=2.6e-3 | 0.91 (0.78, 1.05); p=2.1e-1 | 0.82 ( 0.7, 0.96); p=1.2e-2 | 0.83(0.74,0.91);p=2.92E-06 |
| **SNP1 conditional on SNP2 + covs** | 1.16 (0.99, 1.35); p=6.6e-2 | 1.24 (1.08, 1.42); p=2.1e-3 | 1.12 (0.91, 1.37); p=2.8e-1 | **1.19(1.09,1.28);p=3E-04** |
| **SNP2 conditional on SNP1 + covs** | 0.87 (0.76, 0.99); p=4.0e-2 | 0.91 (0.78, 1.05); p=2.1e-1 | 0.85 (0.72, 1.02); p=7.8e-2 | 0.88(0.79,0.97);p=3.42E-03 |
|  |  |  |  |  |
| ***RORA/SMAD3 region*** SNP1 = rs11071559 (in *RORA*) & coded allele is T SNP2 = rs744910 (in *SMAD3*) & coded allele is G *Distance=6,376,797* | | | | |
|  | ***FINRISK, H2000, HBC, YFS*** | ***FraminghamHS*** | ***NFBC66*** | ***pooled*** |
| **Frequency of coded allele of SNP1** | 15.53% | 13.53% | 16.07% | 14.86% |
| **Frequency of coded allele of SNP2** | 50.81% | 48.52% | 52.21% | 49.76% |
| **SNP1 + covs** | 0.86 (0.72, 1.03); p=9.7e-2 | 0.89 (0.75, 1.06); p=1.8e-1 | 0.81 (0.65, 1.01); p=6.1e-2 | **0.86(0.75,0.97);p=6.29E-03** |
| **SNP2 + covs** | 1.12 (1.00, 1.27); p=5.8e-2 | 1.06 (0.95, 1.19); p=3.0e-1 | 1.06 (0.91, 1.24); p=4.5e-1 | 1.08(1.01,1.16);p=3.3E-02 |
| **SNP1 conditional on SNP2 + covs** | 0.86 (0.72, 1.03); p=9.8e-2 | 0.89 (0.75, 1.06); p=2.0e-1 | 0.81 (0.65, 1.01); p=5.6e-2 | **0.86(0.75,0.97);p=6.09E-03** |
| **SNP2 conditional on SNP1 + covs** | 1.12 (1.00, 1.27); p=5.8e-2 | 1.06 (0.95,1.17);p=3.0e-1 | 1.07 (0.92, 1.24); p=4.0e-1 | 1.1(1.01,1.2);p=4.5E-02 |
