# Supplementary material for: Genome-Wide Association Studies of Asthma in Population-Based Cohorts Confirm Known and Suggested Loci and Identify an Additional Association near HLA
Source: PLoS One. 2012 Sep 28;7(9):e44008. doi: 10.1371/journal.pone.0044008 (PMC3461045; doi:10.1371/journal.pone.0044008)
Supplement: Table S4 — Variance explained by the GWAs hits under a liability threshold model. (DOCX) [file pone.0044008.s008.docx]

**Table S4:** Variance explained by the GWAs hits under a liability threshold model.

| **LOCUS** | **SNP** | **allele** | **Frequency in APCAT** | **Odds Ratio in APCAT** | **Variance Explained** | **Used for calculating the total** |
| --- | --- | --- | --- | --- | --- | --- |
| GSDMA | rs3894194 | A | 0.4234 | 1.110 | 0.137% | yes |
| GSDMB | rs2305480 | A | 0.4822 | 0.940 | 0.048% | no |
| ORMDL3 | rs7216389 | T | 0.4729 | 1.110 | 0.139% | yes |
| IL33 | rs3939286 | T | 0.2511 | 1.180 | 0.267% | yes |
| IL33 | rs1342326 | C | 0.1662 | 1.180 | 0.197% | no |
| HLA-DQ | rs9273349 | C | 0.5005 | 1.220 | 0.511% | yes |
| IL1RL1 | rs1420101 | T | 0.3493 | 1.160 | 0.259% | yes |
| IL18R1 | rs3771166 | A | 0.3977 | 0.890 | 0.164% | no |
| SMAD3 | rs744910 | A | 0.4976 | 0.920 | 0.088% | yes |
| IL2RB | rs2284033 | A | 0.4741 | 0.980 | 0.005% | yes |
| IL13 | rs1295686I | C | 0.6938 | 0.900 | 0.119% | yes |
| DENND1B | rs2786098 | T | 0.2054 | 0.930 | 0.043% | yes |
| PDE4D | rs1588265 | G | 0.3035 | 0.960 | 0.018% | yes |
| **Total Variance Explained** |  |  |  |  | **1.586%** |  |

The variance explained was calculated using a liability threshold model by So et al (Genetic Epidemiology, 2011) assuming an asthma prevalence of 9% (the prevalence in APCAT). Only the best SNP from each locus is used to calculate the total variance explained.
