# Supplementary material for: Genome-Wide Association Studies of Asthma in Population-Based Cohorts Confirm Known and Suggested Loci and Identify an Additional Association near HLA
Source: PLoS One. 2012 Sep 28;7(9):e44008. doi: 10.1371/journal.pone.0044008 (PMC3461045; doi:10.1371/journal.pone.0044008)
Supplement: Table S5 — Stratified association analysis of previously published SNPs and two novel loci emerging from this paper. (DOCX) [file pone.0044008.s009.docx]

**Table S5:** Stratified association analysis of previously published SNPs and two novel loci emerging from this paper.

|  |  |  |  |  |  | ***Smoking stratified analysis*** | | | | | |  | ***Allergic status stratified analysis*** | | | | |
| --- | --- | --- | --- | --- | --- | --- | --- | --- | --- | --- | --- | --- | --- | --- | --- | --- | --- |
| SNP (Gene) | **chr (position)** | **Effect/ Other Allele** | **Effect allele freq.** | **No Stratification** |  | **in Never Smokers** | **in Ex-smokers** | | **in Current Smokers** | **Heterogeneity p-value** | |  | **in Non-allergic Individuals** | **in Allergic Individuals** | | **Heterogeneity p-value** | |
| ***Previously reported asthma risk variants*** | | | | | | | | | | | | | | | | | |
| rs3894194 (GSDMA) | 17 (35375519) | A/G | 41.7% | 1.11 (1.03, 1.19); p=2.4e-03 |  | 1.10(0.99,1.22); p=4.10E-02 | 1.15 (1.03,1.27); p=1.3E-02 | | 1.04 (0.89,1.2); p=2.99E-01 | | 0.623 |  | 1.07 (0.93,1.21); p=1.84E-01 | 1.12 (1.02,1.22); p=1.16E-02 | | 0.570 | |
| rs2305480 (GSDMB) | 17 (35315722) | A/G | 51.0% | 0.94 (0.87,1.01); p=4.1E-02 |  | 1.00 (0.89,1.11) p=5.10E-01 | 0.87 (0.75,0.99); p=1.3E-02 | | 0.92 (0.77,1.07); p=1.34-01 | | 0.242 |  | 1.01 (0.87,1.15); p=5.72E-01 | 0.90 (0.8,1.0); p=1.99E-02 | | 0.186 | |
| rs7216389 (ORMDL3) | 17 (35323475) | T/C | 46.6% | 1.11 (1.04,1.19); p=2.1E-03 |  | 1.06 (0.94,1.18); p=1.60E-01 | 1.20 (1.08,1.33); p=2.1E-03 | | 1.10 (0.94,1.26); p=1.19E-01 | | 0.340 |  | 1.05 (0.9,1.2); p=2.73E-01 | 1.17 (1.06,1.27); p=1.53E-03 | | 0.245 | |
| rs3939286 (IL33) | 9 (6200099) | T/C | 25.1% | 1.18 (1.10,1.26); p=4.8E-05 |  | 1.18 (1.05,1.31); p=6.40E-03 | 1.10 (0.95,1.25); p=1.0E-01 | | 1.33 (1.16,1.5); p=4.66E-04 | | 0.242 |  | 1.27 (1.11,1.43); p=1.43E-03 | 1.20 (1.09,1.32); p=7.75E-04 | | 0.587 | |
| rs1342326 (IL33) | 9 (6180076) | C/A | 16.4% | 1.18 (1.09,1.28); p=2.5E-04 |  | 1.22 (1.07,1.37); p=5.40E-03 | 1.05 (0.88,1.22); p=2.9E-01 | | 1.33 (1.14,1.53); p=1.99E-03 | | 0.175 |  | 1.33 (1.15,1.52); p=1.20E-03 | 1.15 (1.02,1.29); p=2.00E-02 | | 0.218 | |
| rs9273349^a^ (HLA-DQ) | 6 (32733847) | C/T | 50.1% | 1.22 (1.07,1.38); p=5.6E-03 |  | 1.12 (0.87,1.37); p=1.90E-03 | 1.34 (1.06,1.62); p=2.0E-01 | | 1.19 (0.91,1.48); p=1.14E-01 | | 0.637 |  | 1.25 (0.92,1.58); p=9.11E-02 | 1.30 (1.11,1.49); p=3.31E-03 | | 0.853 | |
| rs3771166 (IL18R1) | 2 (102352654) | A/G | 60.1% | 0.89 (0.82,0.97); p=2.5E-03 |  | 0.96 (0.84,1.07); p=2.20E-01 | 0.87 (0.75,1); p=1.8E-02 | | 0.82 (0.67,0.97); p=4.50E-03 | | 0.262 |  | 0.82 (0.67,0.97); p=4.26E-03 | 0.95 (0.84,1.06); p=1.85E-01 | | 0.109 | |
| rs1420101 (IL1RL1) | 2 (102324148) | T/C | 34.4% | 1.16 (1.08,1.23); p=9.9E-04 |  | 1.09 (0.98,1.21); p=6.70E-02 | 1.14 (1.01,1.27); p=2.6E-02 | | 1.32 (1.17,1.46); p=1.28E-04 | | 0.136 |  | 1.18 (1.03,1.33); p=1.43E-02 | 1.13 (1.02,1.23); p=1.16E-02 | | 0.625 | |
| rs744910 (SMAD3) | 15 (65233839) | A/G | 50.6% | 0.92 (0.85,1.00); p=1.7E-02 |  | 0.97 (0.86,1.07); p=2.60E-01 | 0.89 (0.76,1.02); p=3.5E-02 | | 0.93 (0.78,1.08); p=1.68E-01 | | 0.619 |  | 0.98 (0.84,1.11); p=3.63E-01 | 0.96 (0.86,1.06); p=2.24E-01 | | 0.877 | |
| rs11071559 (RORA) | 15 (58857280) | T/C | 85.0% | 0.86 (0.75,0.97); p=3.1E-03 |  | 0.9 (0.74,1.07); p=1.1E-01 | 0.91 (0.73,1.09); p=1.4E-02 | | 0.77 (0.54,0.99); p=1.13E-02 | | 0.463 |  | 1.00 (0.81,1.2); p=5.17E-01 | 0.79 (0.64,0.94); p=1.24E-03 | | **0.059** | |
| rs2284033 (IL2RB) | 22 (35863980) | A/G | 51.9% | 0.98 (0.91,1.06); p=3.1E-01 |  | 0.96 (0.84,1.07); p=2.40E-01 | 0.98 (0.85,1.11); p=3.7E-01 | | 1.06 (0.9,1.21); p=7.56E-01 | | 0.608 |  | 0.91 (0.78,1.05); p=1.01E-01 | 1.10 (1.0,1.2); p=9.62E-01 | | **0.038** | |
| rs1295686 (IL13) | 5 (132023742) | C/T | 30.5% | 0.90 (0.82,0.98); p=5.4E-03 |  | 0.91 (0.78,1.03); p=6.30E-02 | 0.87 (0.72,1.02); p=3.0E-02 | | 0.97 (0.79,1.14); p=3.46E-01 | | 0.658 |  | 0.88 (0.72,1.03); p=5.20E-02 | 0.91 (0.80,1.02); p=5.44E-02 | | 0.694 | |
| rs2244012 (RAD50) | 5 (131929124) | G/A | 23.3% | 1.05 (0.96,1.14); p=1.3E-01 |  | 0.95 (0.81,1.08); p=2.06E-01 | 1.08 (0.93,1.22); p=1.7E-01 | | 1.04 (0.86,1.22); p=3.40E-01 | | 0.958 |  | 1.08 (0.91,1.25); p=1.86E-01 | 0.99 (0.87,1.11); p=5.40E-01 | | 0.432 | |
| rs2073643 (SLC22A5) | 5 (131751187) | C/T | 52.8% | 0.96 (0.88,1.04); p=1.6E-01 |  | 0.98 (0.86,1.1); p=3.50E-01 | 0.94 (0.81,1.07); p=1.7E-01 | | 0.97 (0.82,1.13); p=3.70E-01 | | 0.892 |  | 0.89 (0.74,1.05); p=7.47E-02 | 0.98 (0.87,1.08); p=3.23E-01 | | 0.353 | |
| rs2786098 (DENND1B) | 1 (195592531) | T/G | 79.8% | 0.93 (0.89,1.02); p=5.8E-02 |  | 1.01 (0.88,1.14); p=5.60E-01 | 0.9 (0.74,1.06); p=9.1E-02 | | 0.88 (0.7,1.07); p=9.48E-02 | | 0.385 |  | 0.95 (0.77,1.12); p=2.65E-01 | 0.94 (0.81,1.07); p=1.74E-01 | | 0.962 | |
| rs1588265^b^ (PDE4D) | 5 (59405551) | G/A | 70.1% | 0.96 (0.85,1.06); p=2.0E-01 |  | 0.84 (0.71,0.97); p=3.60E-03 | 0.98 (0.85,1.12); p=4.1E-01 | | 0.92 (0.76,1.09); p=1.72E-01 | | 0.248 |  | 0.82 (0.66,0.99); p=1.02E-02 | 0.94 (0.83,1.06); p=1.64E-01 | | 0.181 | |
|  | | | | | | | | | | | | | | | | | |
| ***Risk variants emerging from this paper*** | | | | | | | | | | | | | | | | | |
| rs13408661 (*IL1RL1 /IL18R1*) | 2 (102321514) | G/A | 0.84% | 1.29 (1.18,1.4); p=3.80E-06 |  | 1.28 (1.27,1.47); p=1.41E-03 | | 1.23 (1.05,1.41); p=1.24E-02 | 1.42 (1.19,1.65); p=1.26E-03 | | 0.554 |  | 1.39 (1.18,1.60); p=1.03E-03 | | 1.20 (1.05,1.32); p=7.17E-03 | | 0.738 |
| rs9268516 (HLA Region) | 6 (32487467) | T/C | 0.24 | 1.26 (1.17,1.35); p=1.30E-07 |  | 1.22 (1.09,1.36); p=1.30E-03 | | 1.49 (1.27,1.71); p=8.7E-05 | 1.19 (1.02,1.36); p=2.06E-02 | | 0.425 |  | 1.27 (1.11,1.43); p=1.58E-03 | | 1.23 (1.11,1.35); p=4.32E-04 | | 0.102 |

All the p-values presented in this table are based on one-sided testing except in the no stratification column. ^a^ SNP rs9273349 is present in NFBC66 data set only. ^b^Results exclude the Framingham Heart Study, which contributed to the original report in Himes et al (2009) (E[23](#_ENREF_23)).
